# A Statistical Learning Framework for Materials Science: Application to Elastic Moduli of *k*-nary Inorganic Polycrystalline Compounds

**DOI:** 10.1038/srep34256

**Published:** 2016-10-03

**Authors:** Maarten de Jong, Wei Chen, Randy Notestine, Kristin Persson, Gerbrand Ceder, Anubhav Jain, Mark Asta, Anthony Gamst

**Affiliations:** 1 Department of Materials Science and Engineering, University of California, Berkeley, Berkeley, CA 94720, USA; 2 Energy Technologies Area, Lawrence Berkeley National Laboratory, Berkeley, CA 94720, USA; 3 Computational and Applied Statistics Laboratory, San Diego Supercomputer Center, University of California, San Diego, La Jolla, CA 92093, USA; 4 Materials Sciences Division, Lawrence Berkeley National Laboratory, Berkeley, CA 94720, USA

## Abstract

Materials scientists increasingly employ machine or statistical learning (SL) techniques to accelerate materials discovery and design. Such pursuits benefit from pooling training data across, and thus being able to generalize predictions over, *k*-nary compounds of diverse chemistries and structures. This work presents a SL framework that addresses challenges in materials science applications, where datasets are diverse but of modest size, and extreme values are often of interest. Our advances include the application of power or Hölder means to construct descriptors that generalize over chemistry and crystal structure, and the incorporation of multivariate local regression within a gradient boosting framework. The approach is demonstrated by developing SL models to predict bulk and shear moduli (*K* and *G*, respectively) for polycrystalline inorganic compounds, using 1,940 compounds from a growing database of calculated elastic moduli for metals, semiconductors and insulators. The usefulness of the models is illustrated by screening for superhard materials.

In recent years, first-principles methods for calculating properties of inorganic compounds have advanced to the point that it is now possible, for a wide range of chemistries, to predict many properties of a material before it is synthesized in the lab^1^. This achievement has spurred the use of high-throughput computing techniques^2^,^3^,^4^,^5^ as an engine for the rapid development of extensive databases of calculated material properties^6^,^7^,^8^,^9^,^10^,^11^,^12^. Such databases create new opportunities for computationally-assisted materials discovery and design, providing for a diverse range of engineering applications with custom tailored solutions. But even with current and near-term computing resources, high-throughput techniques can only analyze a fraction of all possible compositions and crystal structures. Thus, statistical learning (SL), or machine learning, offers an express lane to further accelerate materials discovery and inverse design^2^,^5^,^13^,^14^,^15^,^16^,^17^,^18^,^19^,^20^,^21^,^22^,^23^,^24^,^25^,^26^,^27^. As statistical learning techniques advance, increasingly general models will allow us to quickly screen materials over broader design spaces and intelligently prioritize the high-throughput analysis of the most promising material candidates.

One encounters several challenges when applying SL to materials science problems. Although many elemental properties are available, we typically do not know how to construct optimal *descriptors* for each property, over a variable number of constituent elements. For instance, if one believes that some average of atomic radii is an important descriptor, there are many different averages, let alone possible weighting schemes, that one might investigate. This challenge may be reduced by placing restrictions on the number of constituent elements or types of chemistries or structures considered, but such restrictions reduce the generalizability of the learned *predictor*. Materials science datasets are often also smaller than those available in domains where SL has an established history. This requires that SL be applied with significant care in order to prevent *over-fitting* the model. Over-fitting leads to predictions that are less generalizable to new data than anticipated^28^, such that predictions are less accurate than expected. At the same time, smaller datasets challenge us to use the available data as wisely as possible. This may include leveraging observations related to the smoothness of the underlying physical phenomenon, and the use of an appropriate risk criterion, rather than partitioning the available data into distinct training and test sets. For SL to have the greatest impact on materials discovery and design, we must pursue techniques that make maximal use of the available data. This requires approaches that are capable of systematically pooling training data across, and are thus capable of generalizing predictions over, *k*-nary compounds of diverse chemistries and structures.

The successful application of SL requires the selection of an appropriate set of descriptor candidates. In materials science problems, the candidates must be capable of both “uniquely characterizing”^22^ a diverse range of compounds, and sufficiently explaining the diversity of the phenomenon being learned. Thus, the selection of descriptor candidates is a crucial and active field of investigation within materials science (e.g. refs 14, 22 and 23), as the field endeavors to develop general models with high predictive accuracy. Previous work in materials science has included both *categorical* descriptors (e.g. refs 23 and 24) and *continuous* descriptors (e.g. refs 22 and 23). Although both types of descriptors may be legitimately used in SL, special care should be taken when using categorical descriptors, as each such descriptor essentially (i.e., unless there is sufficient smoothing across cells) partitions the space of compounds into disjoint cells, which quickly increases the degrees of freedom and thus the risk of over-fitting the model.

SL applications should always include descriptor candidates suggested by known, scientifically relevant relationships^22^,^23^. But in order to construct models that accurately generalize across diverse datasets, such candidates will typically need to be augmented with additional descriptor candidates, capable of bridging across the simplifying assumptions that divide less generalizable models. Without these additional candidates, attempts to learn more general models will be stifled, as it will be impossible to discover new, unexpected relationships. Here we introduce the use of Hölder means, also known as generalized or power means, as an ordered approach to explicitly constructing descriptor candidates from variable length numeric lists. Hölder means describe a family of means that range from the minimum to maximum functions, and include the harmonic, geometric, arithmetic, and quadratic means^29^. This paper advances previous work by constructing descriptor candidates as Hölder means, which, to the best of our knowledge, has not previously been done in the field of materials science.

Having discussed the construction of descriptor candidates, we now introduce gradient boosting machine local polynomial regression (GBM-Locfit), which is a SL technique that we developed to leverage the available data as wisely as possible. Energy minimization problems often enforce smoothness in the functions mapping useful descriptors to outcomes. Statistical learning techniques may exploit such smoothness, when present, in order to produce models that are as accurate as possible for a fixed amount of training data; such considerations are more important when working with smaller training datasets than with larger datasets. GBM-Locfit utilizes multivariate local polynomial regression, as implemented in Locfit^30^, within a gradient boosting machine (GBM) framework^31^. Local polynomial regression performs a series of weighted regressions within a moving window, with a weight function that gives greatest weight to observations near the center of the window, producing a smooth curve that runs through the middle of the observations^32^,^33^. GBM uses a gradient descent algorithm to iteratively assemble a predictor while minimizing an appropriate, typically squared error, loss function^31^. Our approach enforces more smoothness in the functions mapping descriptors to outcomes than traditional tree-based GBM methods, which we suggest is appropriate for this and many other materials science problems. Additionally, the enforced smoothness helps minimize boundary bias (i.e., when the solution is flat over some peripheral region of the space of descriptors), which can be problematic with tree-based techniques when the data has sparsely populated tails. We believe GBM-Locfit will be advantageous for many materials science problems where datasets are of modest size, the underlying physical phenomenon is reasonably smooth and sparse regions have been carefully studied and are of particular interest.

To illustrate both our GBM-Locfit approach and the use of descriptor candidates constructed as Hölder means, we predict the elastic bulk and shear moduli (*K* and *G*, respectively) of *k*-nary inorganic polycrystalline compounds. These moduli govern the stress-strain relations of isotropic materials within the linear-elastic range and are central to governing the mechanical behavior of materials in diverse contexts spanning geophysics to structural engineering. In addition, elastic constants are known to correlate with a wide range of other materials properties, including ductility and hardness^34^,^35^,^36^,^37^ and thermal conductivity^38^,^39^,^40^. Further, the single-crystal elastic constants are a direct measure of the bonding strength and directionality in a material, and are thus widely employed in the development of theoretical models for interatomic forces. Due to the importance of these properties, extensive efforts have been devoted to developing theoretical models of elastic moduli, relating their magnitude to structural and electronic properties such as atomic density, coordination, cohesive energy and Fermi energy^27^,^41^,^42^,^43^,^44^,^45^,^46^. But all of these models consider specific subsets of chemistries or structures, limiting their use for predicting the elastic properties of a diverse range of materials. A recent investigation employing nonparametric regression^24^ and categorical descriptors considered elastic constants for a diverse range of materials, but the results fail to generalize to new data (see Supplementary Figures S6 and S7). In this paper, we demonstrate the application of the SL framework described above to develop broadly applicable models for *K* and *G*, expressed in terms of a few descriptors that are either currently tabulated or easily computed. We demonstrate how such models can be used to enable materials discovery, by screening the hardness of over 30,000 compounds to identify superhard inorganic compounds that are thermodynamically stable or weakly metastable at a temperature of 0 K. Our training dataset consists of 1,940 inorganic compounds from the Materials Project’s growing database of elastic constants constructed using first-principles, quantum mechanical calculations based on Density Functional Theory (DFT)^12^.

The outline of this paper is as follows. In the methods section, we detail our SL framework, which includes safeguards against over-fitting our models. Then the predictive models for the elastic moduli are described in the results section, including an overview of the descriptors and the prediction accuracy. In addition, we present a screening process for superhard materials and present a DFT validation. In the discussion section, we examine known issues with the accuracy of the predictions and conclude with a summary of the main advances presented in this work.

## Methods

We begin our methods section with some background on local polynomial regression, which was introduced to the statistics literature by Stone^32^ and Cleveland^33^. Loader^30^ provides a general, yet thorough discussion of local regression, as well as implementation details of the Locfit software. Simply put, multivariate local regression produces a smooth surface that runs nominally through the middle of the observations. Thus, given response variables, *y*, and predictor variables, *x*, Locfit estimates a regression function, *η*, which relates these quantities, where 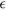 is noise (assumed to be independent and identically distributed with zero mean and finite variance):


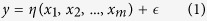


Globally, no strong assumptions are made concerning the form of *η*, the underlying function being estimated. Locally, at each fitting point, the underlying function is assumed to be smooth, such that Taylor’s theorem allows the behavior to be described with a low order polynomial. Specifically, a once-differentiable function can be approximated locally by a linear function, and more generally, a *k*-times differentiable function can be approximated locally by a *k*th-order polynomial. In order to make local estimates of *η*, one must select a bandwidth, or smoothing window, and an appropriate smoothing kernel or weight function. Appropriate weight functions, such as Locfit’s default tricubic weight function, give greatest weight to observations near the center of the smoothing window and zero weight to observations outside the window. The local estimate of *η*, at each fitting point, is the intercept of the local regression centered at the fitting point, and these local estimates combine to produce a smooth estimate of the underlying function. We are interested in estimating smooth functions, because energy minimization problems often enforce smoothness in the functions mapping useful descriptors to outcomes.

In this paper, we distinguish between *composition* and *structural* descriptors. Composition descriptors, such as average atomic radius and weight, are calculated from elemental properties and only require knowledge of a compound’s composition. Structural descriptors, such as cohesive energy and volume per atom, require knowledge of a compound’s specific structure (in addition to composition), and may be determined experimentally or calculated using DFT. We seek composition descriptors that generalize over *k*-nary compounds, but do not have *a priori* knowledge of how to combine the various elemental properties to construct descriptors that are optimal for our specific, yet very general problem. Thus we construct composition descriptors as a series of weighted Hölder means, rely upon Locfit to capture any necessary non-linearities, and rely upon model selection techniques and our GBM framework to select which descriptors are most useful at each iteration, and for each specific problem. Because GBM implements a version of the least absolute shrinkage and selection operator (LASSO)^47^, our approach has similarities to the statistical learning approach advocated by Ghiringhelli *et al*.^22^, but is less reliant upon sufficient, *a priori*, scientific insight and may thus be applied to more general problems.

In equation (2), *μ*_*p*_(*x*) represents the Hölder mean *μ*, to the power *p*, of the property *x*, taken over *i* values, with associated weights *w*_*i*_^29^. Equation (3) gives the Hölder mean when *p* equals zero. Hölder means describe a family of generalized means that range from the minimum function (when *p* = −∞) to the maximum function (*p* = ∞). An example would be calculating the cubic mean (*p* = 3) of atomic radii over all constituent elements in a particular composition, where the weights would be the molar quantities of each element.


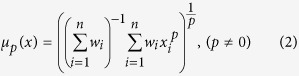



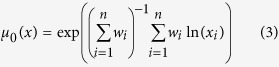


In this work, we only consider the Hölder means with integer power values between negative and positive four, which include the well known harmonic mean (*p* = −1), geometric mean (*p* = 0), arithmetic mean (*p* = 1), and quadratic or Euclidean mean (*p* = 2). We construct these Hölder based composition descriptors for each of eight elemental properties listed in Table 1 (upper), using elemental properties from pymatgen^48^. We also consider the structural descriptors listed in Table 1 (lower); most of these descriptors are obtained directly or post-processed from a single density functional theory (DFT) calculation per compound. The cohesive energy per atom, *E*_*c*_, is estimated from DFT by subtracting the atom-in-a-box energies of the constituent elements, from the formation energy of each compound. Following a Voronoi tessellation^49^ of each unit cell, atomic coordinations, nearest-neighbor bond lengths, and bond angles between adjacent neighbors are calculated for each site. Additional structural descriptors are then constructed as Hölder means of these quantities over all sites; please see Supplementary Table SI for a full list of all investigated descriptors.

Our GBM-Locfit implementation uses established model selection techniques, including 10-fold cross-validation and a conservative risk criterion, to determine which descriptors are the most useful for predicting *K* and *G*, without over-fitting the training data. The GBM framework iteratively assembles a predictor, *P*, as a sum of Locfit smoothed *weak learners*, *η*_*i*_. At each iteration, GBM selects the smoothed weak learner candidate that leads to the greatest reduction in the size of the residual, 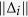, but attenuates each weak learner by the *learning rate*, *λ*, as shown in equation (4). Both of our final models use a learning rate of 5% and limit the level of interaction to 3 descriptors, *D*_*j*_, meaning that Locfit is only run with three descriptors at a time, to create each smoothed weak learner candidate.


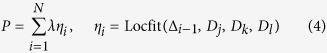


Although it is possible that the number of iterations required to achieve a given prediction error could be reduced by tuning Locfit’s smoothing parameters, we have opted to use Locfit’s default smoothing parameters and rely on the GBM method to provide an appropriate amount of flexibility to the fitted model. The one exception to this, is that Locfit’s degree of local polynomial is set to linear for all models, rather than using the default setting of quadratic.

**Figure 1:**
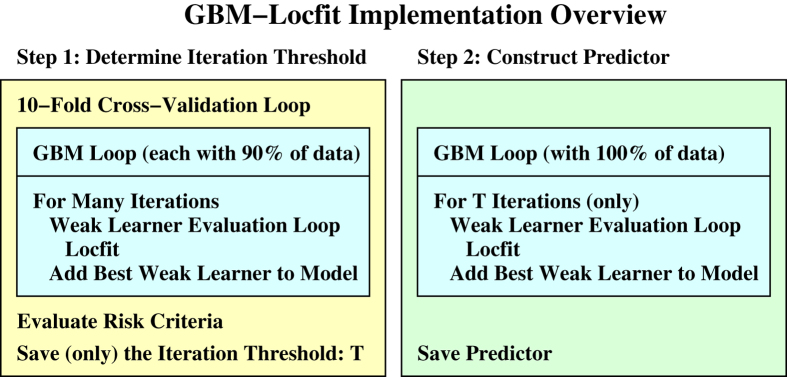
GBM-Locfit implementation consists of two distinct steps. First, the iteration threshold is determined per the risk criterion, by running the GBM loop within a 10-fold cross-validation loop, in order to estimate the prediction mean squared error and associated standard error for each iteration. Second, the final GBM-Locfit model is fit with 100% of the data, while limiting the number of GBM iterations to the iteration threshold. This approach utilizes all of the available data for training, gives equal consideration to each compound, and avoids over-fitting the model to the data.

Our GBM-Locfit implementation utilizes *all* of the available data for training and relies on a conservative risk criterion to limit the number of iterations, rather than an explicitly partitioned test dataset, to avoid over-fitting the model to the data. As summarized graphically in Fig. 1, we perform 10-fold cross-validation (CV), using 90% of the data to select the weak learner that minimizes the squared error loss function and the remaining 10% of the data to estimate the mean and standard deviation of the out-of-sample squared errors, for each iteration and fold. After this process is completed for each fold and for a large number of iterations, the prediction errors are calculated as the mean (over folds) out-of-sample squared error for each iteration, and the standard errors of the prediction errors are estimated from the standard deviations of the out-of-sample squared errors for each iteration. The risk criterion determines the iteration threshold as the first iteration for which the prediction mean squared error (MSE) is less than the sum of the minimum prediction MSE (over all iterations) and the standard error of the prediction MSE at that minimum^50^, which has been shown to be conservative^51^. Please see Supplementary Fig. S1 for example performance curves. A more commonly used, but unconservative risk criterion is to simply establish the iteration threshold as the minimum prediction MSE (without adding one standard error), but this seems overly optimistic, particularly when the sample size is small (relative to the number of descriptor candidates) or the prediction MSE curve lacks a distinct minimum. After the iteration threshold is determined, a final GBM-Locfit model is fit using 100% of the data, but limiting the number of iterations to the previously established iteration threshold, to avoid over-fitting the model to the data. By limiting the number of GBM iterations, we inherently limit the number of weak learners, since each iteration adds one weak learner term to the predictor, as in equation (4).

## Results

**Figure 2:**
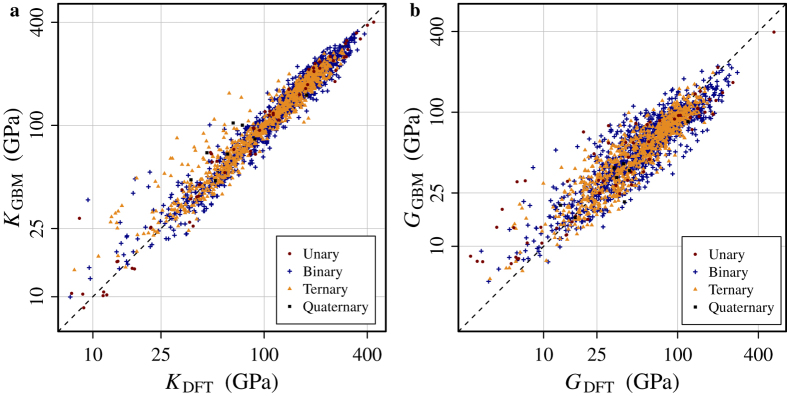
Predictions. Comparison of DFT training data with GBM-Locfit predictions for *K* (**a**) and *G* (**b**). Training set consists of 65 unary, 1091 binary, 776 ternary, and 8 quaternary compounds.

We demonstrate GBM-Locfit by learning the Voigt-Reuss-Hill (VRH) averages^52^ of the elastic bulk and shear moduli (*K* and *G*, respectively) which characterize polycrystalline compounds. More specifically, we learn log(*K*) and log(*G*) to avoid having the squared error loss function severely overweight the higher moduli materials. We present our predictions graphically for *K* and *G* in Fig. 2, by comparing the VRH moduli from our DFT training set^12^ with those learned by our GBM-Locfit method.

Our best four descriptor models for log(*K*) and 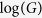 are summarized in Table 2. None of our models with more than four descriptors have significantly better predictive accuracy than these four descriptor models, based on comparisons of prediction mean squared error and their associated standard errors. And all of our models with less than four descriptors have significantly less predictive accuracy.

For both *K* and *G*, the structural descriptors log(*V*), log of volume per atom, and *E*_*c*_, cohesive energy per atom, are very important, with a combined relative influence of 66.0% for *K* and 72.9% for *G*. Notably, these two descriptors are more useful for predicting *K* and *G* than any of the Voronoi based structural descriptors, which were constructed to capture individual attributes of the local environments. Yet the usefulness of these two information-rich descriptors is not surprising, since log(*V*) and *E*_*c*_ incorporate information regarding the local environments, including coordination, bond angles, and bond lengths.

For modulus *X*, relative error is defined as:


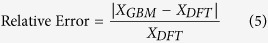


Over half of our predictions have a relative error of less than 10% for *K*, and less than 20% for *G*, as shown in Table 3.

**Figure 3:**
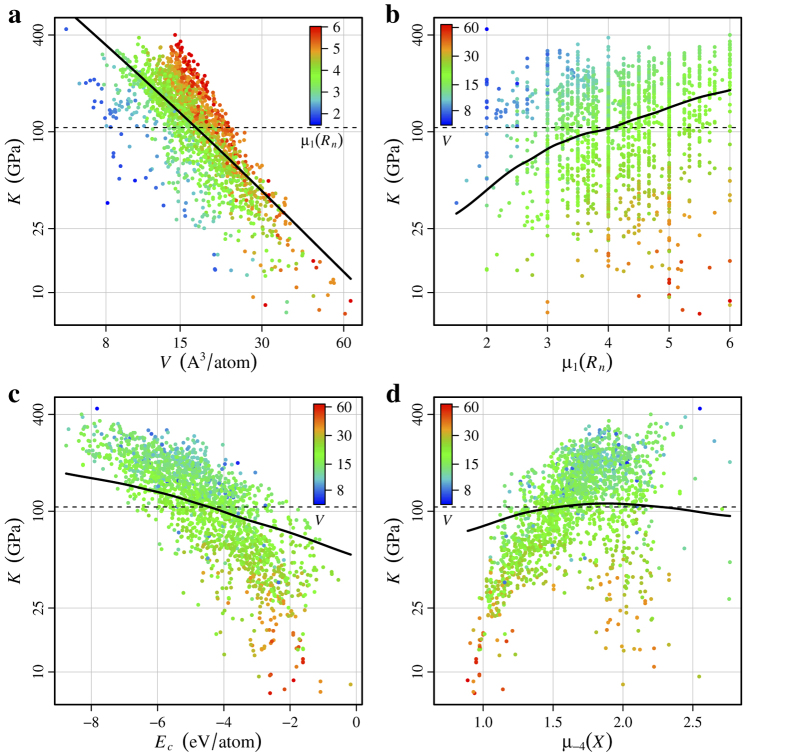
Partial dependence plots for *K*. Partial dependence curves are shown as solid black lines for: (**a**) volume per atom, (**b**) arithmetic mean of elemental row number, (**c**) cohesive energy, and (**d**) quartic-harmonic mean of elemental electronegativity. Training data points are shown in the background, colored per the descriptor indicated below each colorbar, to help illustrate the relationship between descriptors. The mean of the outcome (*K*) is shown as a thin dashed black line for reference.

**Figure 4:**
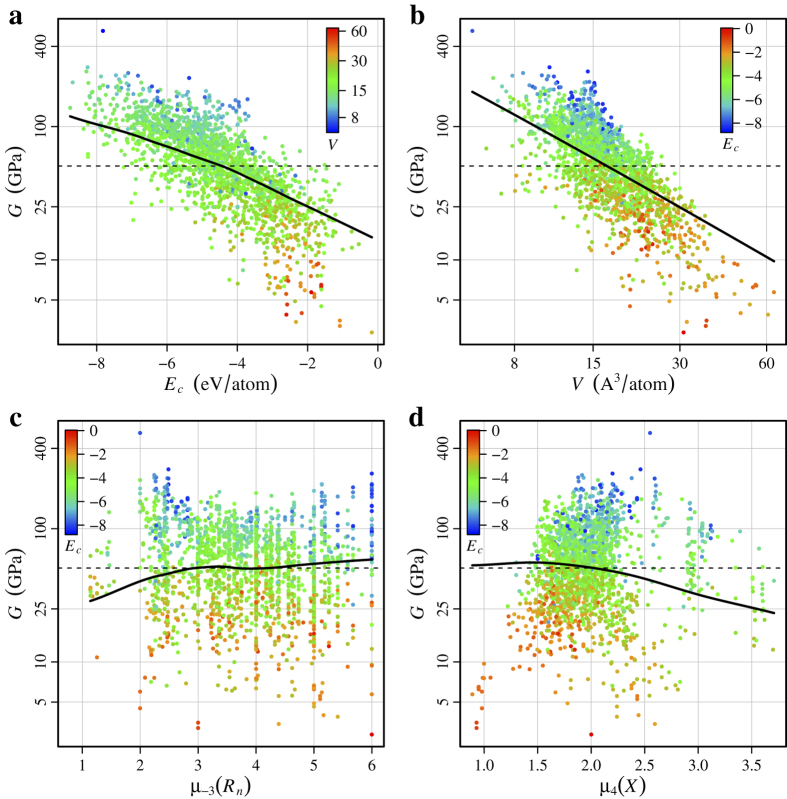
Partial dependence plots for *G*. Partial dependence curves are shown as solid black lines for: (**a**) cohesive energy, (**b**) volume per atom, (**c**) cubic-harmonic mean of elemental row number, and (**d**) quartic mean of elemental electronegativity. Training data points are shown in the background, colored per the descriptor indicated below each colorbar, to help illustrate the relationship between descriptors. The mean of the outcome (*G*) is shown as a thin dashed black line for reference.

Figures 3 and 4 show marginal predictor, or partial dependence, plots for *K* and *G*, which provide a one dimensional summary of the effect of each descriptor on the overall prediction. Marginal predictor summaries account for the effects of the other descriptors^28^, so any correlation between descriptors reduces the predictive influence of one or more of the correlated descriptors. The marginal predictor plots indicate an inverse, nearly linear relationship between log(*V*) and log(*K*), and log(*V*) and log(*G*). This agrees with previous findings for *K*^53^,^54^,^55^,^56^, but our results support that this relationship generalizes beyond the specific material classes previously studied, and also applies approximately to *G*. Additionally, the marginal predictor plots indicate an inverse, gently non-linear relationship between *E*_*c*_ and log(*K*), and *E*_*c*_ and log(*G*). Thus, our models indicate compounds with high *K* and *G* are generally densely packed (low log(*V*)) and strongly bonded (high *E*_*c*_), which agree with both previous findings^53^,^54^,^55^,^56^ and physical intuition. Furthermore, the strong influence of log(*V*) and *E*_*c*_ on *K* and *G*, combined with their similar marginal predictor plots, underscore the strong correlation between the two moduli.

Although *E*_*c*_ is an important predictor of *K*, the composition descriptor *μ*_1_(*R*_*n*_), arithmetic mean of elemental row number, ranks as the second most influential descriptor of *K*. The marginal predictor plot for *μ*_1_(*R*_*n*_) indicates a roughly quadratic relationship with log(*K*), indicating that compounds with a higher arithmetic average of row number generally have a higher *K*. The final descriptor for *K*, with a comparatively small relative influence in our model, is *μ*_−4_(*X*), the quartic-harmonic mean of elemental electronegativity, whose marginal predictor plot indicates that compounds with low average electronegativity generally have a lower *K*. Although this electronegativity descriptor has a small relative influence and fairly weak partial dependence, these are both *after* accounting for the influence of the other descriptors. The Spearman’s rank correlation between *μ*_−4_(*X*) and *K* is approximately 0.50, which is a moderately strong correlation, as evident in Fig. 3(d). Hölder means of *R*_*n*_ and *X* also complete the set of top four descriptors for *G*, although for *G* the most useful Hölder means are *μ*_−3_(*R*_*n*_), the cubic-harmonic mean of elemental row number, and *μ*_4_(*X*), the quartic mean of elemental electronegativity.

The influence of log(*V*) suggests that it may be possible to develop higher moduli materials from existing compounds by filling interstitial sites (to decrease average volume per atom). But the influence of mean elemental row number will at least partially offset the possible improvement, since elements that could be added to interstitial sites (with minimal disruption of the structure) will generally be smaller than the neighboring elements.

### Screening for superhard materials

As an example illustration, we use the SL model to screen for superhard materials. More details and analyses resulting from this application will be presented in a forthcoming article. Here we focus on the main results to illustrate the utility of such a SL model.

The SL predictors developed in this work allow the rapid estimation of *K* and *G* for thousands of compounds, for which the required descriptors may be easily calculated. Additionally, with appropriate caution, these predictors may be plugged into other relationships, to estimate additional material properties that can be expressed as functions of *K* and *G*. As an example, we estimate Vickers hardness, and then screen for superhard materials, defined as those having a hardness exceeding 40 GPa^57^. Vickers hardness is estimated using a recently published model, that has shown good agreement with experimental measurements for both cubic and non-cubic materials^35^,^58^,^59^:


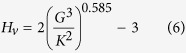


In general, one should exercise caution when plugging results from one or more statistical models into other equations, without an understanding of the error associated with each model and the effect of such errors upon the new outcome. In our case, because the residuals of our SL models for log(*K*) and log(*G*) are positively correlated, the plugin approach should be reasonably accurate when used to generate a relative ranking of hardness. Additionally, we are only using this approach for screening, to identify compounds for more thorough investigation via our DFT workflow.

The screening process for superhard materials starts by considering approximately 30,000 compounds that form a subset of the 66,000 compounds currently in the Materials Project. This subset contains only elements and compounds for which DFT calculations based on the generalized gradient approximation (GGA) are expected to be accurate. In particular, materials containing *f*-electrons or *d*-electrons that require beyond-DFT treatments (such as DFT + U) are not considered in the screening process. This represents a realistic materials-discovery scenario in which screening is carried out on a large number of compounds for which the desired physical property is unknown, either from experiments or calculations.

The SL model is used to estimate the hardness for the 30,000 compounds by employing equation (6). The resulting distribution of hardness values is shown in Supplementary Fig. S8. To further refine the SL predictions of hardness, DFT calculations are performed on the most promising candidates, as identified by the SL model. Consistent with experiments, our SL model identifies diamond as being the hardest compound (among the 30,000 materials considered in the screening), followed by (cubic) boron nitride, a well-known superhard compound^57^. Both of these findings were confirmed by subsequent DFT calculations. Some other compounds that are predicted to be superhard or near-superhard according to the SL model and subsequent DFT calculations are Be_2_C and the family of borides of the form X-B_2_, where X = Ti, Hf, Zr, Sc, Re, V, together with B_4_C. These compounds are all known (near-) superhard materials^60^,^61^. Some compounds have been identified from the SL model (and confirmed by DFT) in this study as (near-) superhard, but are not listed (to the best of our knowledge) in this context in the literature. Such compounds include: Mg(B_6_C)_2_, Sc_2_CrB_6_ and Mg_2_B_24_C, all of which are known compounds that have been synthesized as part of previous investigations^62^,^63^,^64^. Such compounds might provide an interesting starting point for future experimental investigations of superhardness. More details on our screening approach, the DFT calculations and the results will be presented in a forthcoming article.

The hardness screening illustrates the power of using SL models to quickly identify potentially interesting novel materials with target properties. However, the potential use of such SL models reaches far beyond the screening of compounds. In particular, *inverse design* can be performed in which materials that meet a desired requirement are designed computationally by combining a SL model with an optimization routine such as a genetic algorithm. Such methods may be applied not only to search for superhard materials, but also novel thermoelectrics, auxetic materials, photovoltaics or materials with high elastic stiffness, for example. With regards to identifying compounds with high elastic moduli, we note that in the screening process undertaken in this work, several classes of compounds with high *K*, *G* and *K*/*G* are identified using SL and confirmed by DFT. The ratio *K*/*G* is known as Pugh’s ratio and correlates with intrinsic ductility^34^. As with the hardness, DFT calculations are performed on the compounds with the highest value for the property of interest as predicted by SL. The systems that are subsequently investigated by DFT because of promisingly high elastic moduli or *K*/*G* ratio are shown in Supplementary Table SIII. The top-performing candidates in terms of *K*, *G* and *K*/*G* are shown in Supplementary Tables SIV, SV and SVI, respectively. The elastic moduli predicted by both the SL model and the subsequent DFT calculations are tabulated. The calculated *K* and *G* for the systems in Supplementary Table SIII are shown graphically in Supplementary Figures S9 and S10, respectively.

## Discussion

Discrepancies between the DFT and GBM moduli may be caused by (i) shortcomings in our *K* and *G* predictors or (ii) DFT methods-related errors and approximations, which add noise to the underlying physical phenomenon that we are trying to learn. More specifically, predictor shortcomings may include having insufficient training data in some important regions of the space of descriptors, having overlooked relevant descriptor candidates, and any difficulties our GBM-Locfit regressions may have fully capturing the underlying physical behavior. The inorganic polycrystalline compounds in our training set include metallic, ionic, and covalent bonds, but 81% of the compounds qualify as metallic, based on having a DFT-calculated band gap of 0.2 eV or less. Although DFT-calculated band gaps are not entirely reliable, it seems unlikely that a more detailed analysis would result in a meaningfully different characterization of our training set. So while our goal is to predict *K* and *G* for a wide variety of inorganic polycrystalline compounds, regardless of bond details, we acknowledge that our sample is skewed towards metallic compounds. Additionally, we have excluded compounds with *f*-block elements from our training set, since less than 60 such compounds with elastic moduli were available from the Materials Project, and these were insufficient to capture the additional complexities associated with the bonding in such compounds. So although our learned models may be used to predict *K* and *G* for any *k*-nary compound, the reported accuracies may not generalize to compounds with *f*-block elements.

Our GBM prediction errors are larger for *G* than for *K*, particularly for compounds with elastically anisotropic crystalline structures. For these compounds, the Voigt and Reuss bounds are further apart which leads to more uncertainty in the VRH average, so a descriptor of crystal anisotropy would likely improve the model’s predictive accuracy. Other cases where the prediction error is often larger include systems with local magnetic moments, e.g., transition-metal oxides and intermetallic compounds containing Cr, Fe, Co, Mn and Ni. Calculating *K* and *G* for such compounds using DFT is a challenge due to the degrees of freedom associated with magnetic ordering^65^. On the other hand, the GBM-Locfit models lack (explicit) descriptors to include magnetism and might therefore not be able to capture these features accurately. DFT is known to sometimes yield inaccurate cohesive energies, volume per atom and bulk moduli for late transition metals such as Ag and Au but also various metals such as Hg, Cd, Ga, Tl, Pb and Bi^65^. This has been attributed to problems with the description of *d*-electron correlation^66^,^67^,^68^, dispersion^69^, relativistic effects^70^ and spin-orbit coupling in DFT. This is especially a problem in high throughput DFT-calculations, where it is impractical to tune all parameters to yield optimum results for each compound.

## Summary and Conclusions

We have demonstrated our novel GBM-Locfit SL technique and descriptor candidates constructed as Hölder means by predicting the elastic bulk and shear moduli (*K* and *G*, respectively) of *k*-nary inorganic polycrystalline compounds. Our SL framework combines GBM-Locfit (multivariate local regression within a gradient boosting framework), 10-fold cross-validation with a conservative risk criterion, and a diverse set of composition and structural descriptors, which generalize over *k*-nary compounds. Thus, following a single DFT run to determine log(*V*) and *E*_*c*_, predictions for *K* and *G* may be made for any inorganic polycrystalline *k*-nary compound. In fact, the *K* and *G* predictors described here, are already available on the Materials Project website, for all materials for which the full elastic tensors have not yet been calculated via the DFT elastic constants workflow^12^. Additionally, the Materials Project continues to run the DFT workflow on more compounds, so as the available training set grows, the predictive accuracy of subsequent SL models should improve and the reliable identification of additional predictive descriptors may be possible.

More generally, our SL framework dovetails with the extensive materials properties databases made possible by high-throughput computing techniques. Such databases provide training data for new SL models, which facilitate efficient screening of broader design spaces, which then help focus subsequent high-throughput runs on the most promising candidates.

We believe that our GBM-Locfit approach will be advantageous for many materials science problems, when functions mapping descriptors to outcomes are smooth, as is common in problems governed by energy minimization. Although we have introduced descriptors constructed as Hölder means and GBM-Locfit together, they are independent advancements; descriptors constructed as Hölder means may be used with any SL technique. Hölder means provide an ordered approach to constructing a set of descriptor candidates from variable length numeric lists, and should prove useful for a variety of SL problems.

## Additional Information

**How to cite this article**: De Jong, M. *et al*. A Statistical Learning Framework for Materials Science: Application to Elastic Moduli of *k*-nary Inorganic Polycrystalline Compounds. *Sci. Rep.* **6**, 34256; doi: 10.1038/srep34256 (2016).

## Supplementary Material

Supplementary Information

## Figures and Tables

**Table 1:** Overview of descriptor candidates.

Symbol	Description
*G*_*n*_	group number in periodic table
*M*	atomic mass
*R*	atomic radius (empirical)
*R*_*n*_	row number in periodic table
*T*_*b*_	boiling temperature
*T*_*m*_	melting temperature
*X*	electronegativity
*Z*	atomic number
*E*_*c*_	cohesive energy per atom
*E*_*f*_	formation energy per atom
*E*_*g*_	band gap
*E*_*h*_	energy above hull per atom
*ρ*	density
log(*V*)	log of volume per atom
*V*_*c*_	Voronoi based site coordination
*V*_*l*_	Voronoi based site bond lengths
*V*_*θ*_	Voronoi based site bond angles

Descriptor candidates for both moduli include composition descriptors constructed as Hölder means and geometric and arithmetic standard deviations of eight elemental properties (upper) and structural descriptors from DFT and subsequent post-processing (lower).

**Table 2:** GBM-Locfit model summaries.

Model	Rank	Descriptor	Underlying property	RI (%)
*K*	1	log(*V*)	volume per atom	46.6
	2	*μ*_1_(*R*_*n*_)	row number	24.5
	3	*E*_*c*_	cohesive energy	19.4
	4	*μ*_−4_(*X*)	electronegativity	9.5
*G*	1	*E*_*c*_	cohesive energy	37.0
	2	log(*V*)	volume per atom	35.9
	3	*μ*_−3_(*R*_*n*_)	row number	13.8
	4	*μ*_4_(*X*)	electronegativity	13.3

Descriptor rank and relative influence (RI) for our best four descriptor models for *K* and *G*. Composition descriptors are constructed as Hölder means to the power *p*, *μ*_*p*_(*x*), of property *x*.

**Table 3:** GBM-Locfit prediction accuracy.

Model	Iteration Threshold	Prediction RMSE (log(GPa))	Percent of Predictions within Relative Error of			
			5%	10%	20%	30%
*K*	99	0.0750	33.1	58.4	87.3	94.5
*G*	90	0.1378	13.6	28.8	53.0	73.0

Iteration threshold as determined by cross validation, prediction root mean squared error (RMSE), and percentage of predictions within 5, 10, 20, and 30 percent relative error per equation (5) for our best four descriptor models for *K* and *G*.
