# Supplementary material for: A Statistical Learning Framework for Materials Science: Application to Elastic Moduli of k-nary Inorganic Polycrystalline Compounds
Source: Sci Rep. 2016 Oct 3;6:34256. doi: 10.1038/srep34256 (PMC5046120; doi:10.1038/srep34256)
Supplement: Supplementary Information [file srep34256-s1.pdf]

Supplementary Information for: A Statistical Learning Framework for Materials Science: Application to Elastic Moduli of k-nary Inorganic Polycrystalline Compounds

Maarten de Jong[1,*,†], Wei Chen[2,†], Randy Notestine[3], Kristin Persson[1,2], Gerbrand Ceder[1,4], Anubhav Jain[2], Mark Asta[1,4], Anthony Gamst[3]
[1]*Department of Materials Science and Engineering, University of California, Berkeley, Berkeley, CA 94720, USA*
[2]*Energy Technologies Area, Lawrence Berkeley National Laboratory, Berkeley, CA 94720, USA*
[3]*Computational and Applied Statistics Laboratory, San Diego Supercomputer Center, University of California, San Diego, La Jolla, CA 92093, USA and*
[4]*Materials Science Division, Lawrence Berkeley National Laboratory, Berkeley, CA 94720, USA**

(Dated: September 1, 2016)

* Corresponding author: maartendft@gmail.com; †These authors contributed equally

Supplementary methods details

Ensemble statistical learning (SL) techniques construct a *predictor* from a collection or ensemble of *weak learners*, where each weak learner is a function of either a single *descriptor* or just a few descriptors. Gradient boosting (GB), see equation (S1), is a very flexible technique, which makes few assumptions regarding the form of the solution and iteratively builds a predictor, P, from a series of weak learners, η_i, while minimizing the residual of a suitable loss function, such as squared error [1].

$$P = \sum_{i=1}^{N} \eta_i \tag{S1}$$

Each weak learner is either a single descriptor, D_j, or a function of just a few descriptors, see equation (S2), which limits the level of interaction between descriptors.

$$\eta_i = f(D_j, D_k, D_l) \tag{S2}$$

GB implementations use regularization techniques to reduce the risk of over-fitting, which typically include limiting the number of iterations per some risk criteria [2, 3], limiting the level of interaction between descriptors, and employing shrinkage [4]. At each iteration, the weak learner that causes the greatest reduction in the loss function's residual is selected and added to the model, however, when shrinkage is employed, each new term is attenuated by the *learning rate* [5], λ, as in equation (S3).

$$P = \sum_{i=1}^{N} \lambda\, \eta_i \tag{S3}$$

GB is most commonly implemented with regression trees, as in Friedman's Multiple Additive Regression Tree [1] (MART) approach. Trees are computationally efficient and make very minimal smoothness assumptions [6, 7], but prediction accuracy can suffer in regions where the data are sparse. Tree implementations typically limit the minimum number of observations in terminal nodes for stability reasons, which can cause boundary bias, i.e., the solution is flat over some peripheral region of the space of descriptors, even though a smoother, less localized trend may seem clear. Thus, the additional overhead of non-tree approaches may be warranted when the underlying trends are reasonably smooth and sparse regions have been carefully studied and are of particular interest, as in the upper tails of the K and G distributions, which extend to a hexagonal diamond material in our dataset. We have implemented a gradient boosting machine (GBM) that uses multivariate local regression, as implemented in Locfit [8], rather than regression trees. Locfit enforces smoothness in individual weak learners, which are Locfit regressions of a small number of descriptor candidates upon the current residual. Our novel implementation, which we call GBM-Locfit, provides better prediction accuracy in the sparse, upper tails of the K and G distributions than MART based GBM implementations.

In materials science, descriptors may be classified as either *composition* descriptors, which are calculated from elemental properties, or *structural* descriptors, which require knowledge of a compound's specific structure. We construct composition descriptors as a series of weighted Hölder or power means [9] and allow the GBM framework to select which descriptors are most useful for each problem. In this work, we consider the Hölder means, see equations (S4), (S5) and (S6), with optional weights, w_i, with integer power values, p, between negative and positive four, which include the quartic-harmonic mean (p = -4), cubic-harmonic mean (p = -3), quadratic-harmonic mean (p = -2), harmonic mean (p = -1), geometric mean (p = 0), arithmetic mean (p = 1), quadratic or Euclidean mean (p = 2), cubic mean (p = 3), and the quartic mean (p = 4). Additionally, we consider the unbiased, weighted arithmetic (p = 1) and geometric (p = 0) standard deviations, see equations (S7), (S8) and (S9), which provide a measure of

variation of elemental properties within each compound.

$$\mu_p(x) = \left(\frac{1}{\alpha}\sum_{i=1}^{n} w_i x_i^p\right)^{\frac{1}{p}}, \ (p \neq 0) \tag{S4}$$

$$\mu_0(x) = \exp\left(\frac{1}{\alpha}\sum_{i=1}^{n} w_i \ln(x_i)\right) \tag{S5}$$

$$\alpha = \sum_{i=1}^{n} w_i \tag{S6}$$

$$\sigma_1(x) = \left(\beta \sum_{i=1}^{n} w_i \left(x_i - \mu(x)_{p=1}\right)^2\right)^{\frac{1}{2}} \tag{S7}$$

$$\sigma_0(x) = \exp\left(\beta \sum_{i=1}^{n} w_i \ln\left(\frac{x_i}{\mu(x)_{p=0}}\right)^2\right)^{\frac{1}{2}} \tag{S8}$$

$$\beta = \frac{\sum_{i=1}^{n} w_i}{\left(\sum_{i=1}^{n} w_i\right)^2 - \sum_{i=1}^{n} w_i^2} \tag{S9}$$

Our models for both K and G use the same set of 187 descriptor candidates, which include the 88 composition and 99 structural descriptors listed in Table **SI**.

We use GBM-Locfit to learn $\log(K)$ and $\log(G)$, in order to avoid having GBM's squared error loss function severely overweight the higher moduli materials. The learning performance curves for our best four descriptor models are shown in Fig. **S1**. Using the cross validation results, our conservative risk criteria selects the iteration threshold as the first iteration with a prediction (out-of-sample) mean squared error (MSE) less than the sum of the prediction MSE minimum plus one associated standard error (SE). The observed (in-sample) MSE is also shown for reference in the figures.

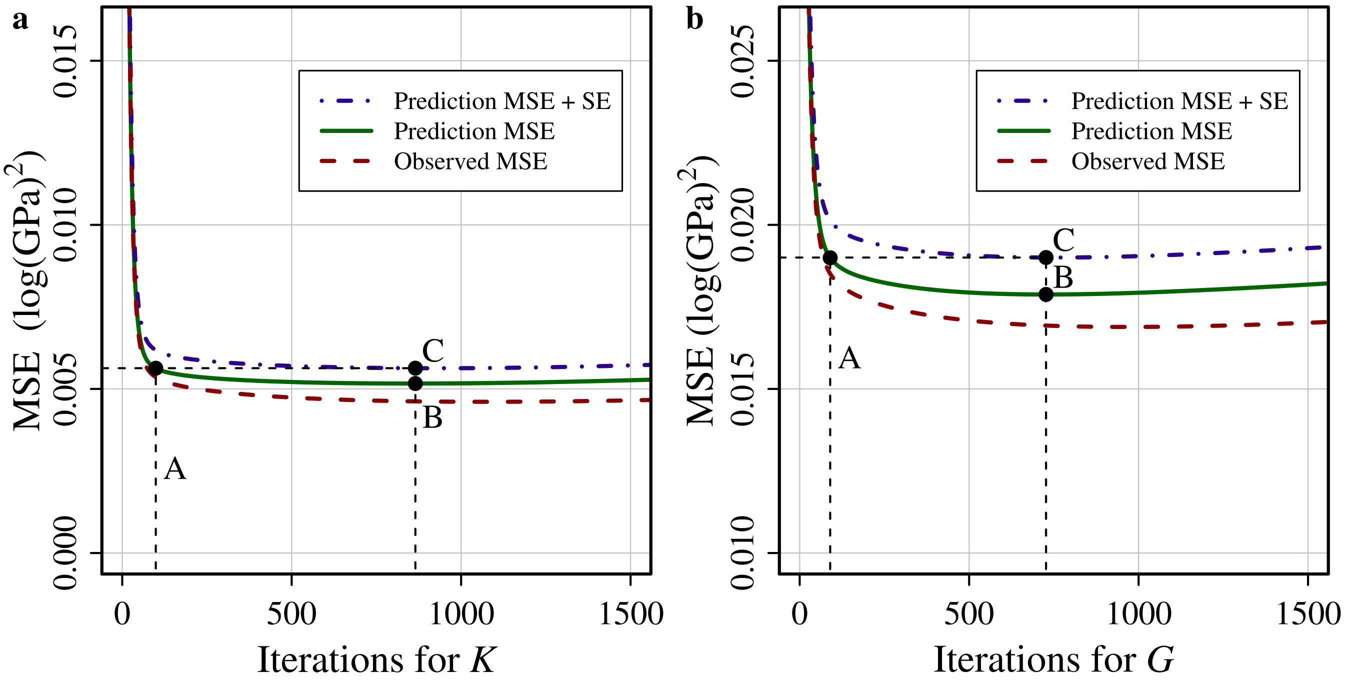

Figure S1: Our risk criteria determines the iteration threshold (line A) of 99 for K (a) and 90 for G (b) as the first iteration with a prediction (out-of-sample) mean squared error (MSE) less than the sum of the prediction MSE minimum (point B) plus one associated standard error (SE; point C). The observed (in-sample) MSE is shown for reference.

The results of our best four descriptor model for $\log(K)$ are presented in Figures **S2** and **S3**. Our best four descriptor model for $\log(G)$ is similarly summarized in Figures **S4** and **S5**. Based on comparisons of prediction mean squared error and their associated standard errors, none of our models with more than four descriptors have significantly better predictive accuracy than these four descriptor models. The chemical formulas and Material Project IDs for the 30 compounds with the largest relative error for K and G are listed in Table **SII**.

TABLE SI: **Detailed list of descriptor candidates.** Descriptor candidates include 88 composition descriptors constructed as Hölder means and deviations of eight elemental properties (upper), six structural descriptors from DFT and subsequent post-processing (middle), and 93 structural descriptors constructed as Hölder means and deviations of 15 Voronoi based site (VBS) quantities (lower), including some absolute difference (AD) and signed difference (SD) quantities. A total of 187 descriptor candidates are considered for each modulus.

Underlying Property	Powers of Hölder Means	Powers of Hölder Devs.	Number of Candidates
Atomic mass	-4, -3, -2, -1, 0, 1, 2, 3, 4	0, 1	11
Atomic number	-4, -3, -2, -1, 0, 1, 2, 3, 4	0, 1	11
Atomic radius (empirical)	-4, -3, -2, -1, 0, 1, 2, 3, 4	0, 1	11
Boiling temperature	-4, -3, -2, -1, 0, 1, 2, 3, 4	0, 1	11
Electronegativity	-4, -3, -2, -1, 0, 1, 2, 3, 4	0, 1	11
Group number in periodic table	-4, -3, -2, -1, 0, 1, 2, 3, 4	0, 1	11
Melting temperature	-4, -3, -2, -1, 0, 1, 2, 3, 4	0, 1	11
Row number in periodic table	-4, -3, -2, -1, 0, 1, 2, 3, 4	0, 1	11
Cohesive energy per atom	N/A	N/A	1
Formation energy per atom	N/A	N/A	1
Band gap	N/A	N/A	1
Energy above hull per atom	N/A	N/A	1
Density	N/A	N/A	1
Log of volume per atom	N/A	N/A	1
VBS coordinations	-4, -3, -2, -1, 0, 1, 2, 3, 4	0, 1	11
VBS mean bond lengths	-4, -3, -2, -1, 0, 1, 2, 3, 4	0, 1	11
VBS mean bond angles	-4, -3, -2, -1, 0, 1, 2, 3, 4	0, 1	11
VBS mean AD of atomic mass	0, 1, 2, 3, 4	1	6
VBS mean SD of atomic mass	1, 2, 4	1	4
VBS mean AD of atomic number	0, 1, 2, 3, 4	1	6
VBS mean SD of atomic number	1, 2, 4	1	4
VBS mean AD of atomic radius	0, 1, 2, 3, 4	1	6
VBS mean SD of atomic radius	1, 2, 4	1	4
VBS mean AD of electronegativity	0, 1, 2, 3, 4	1	6
VBS mean SD of electronegativity	1, 2, 4	1	4
VBS mean AD of group number	0, 1, 2, 3, 4	1	6
VBS mean SD of group number	1, 2, 4	1	4
VBS mean AD of row number	0, 1, 2, 3, 4	1	6
VBS mean SD of row number	1, 2, 4	1	4

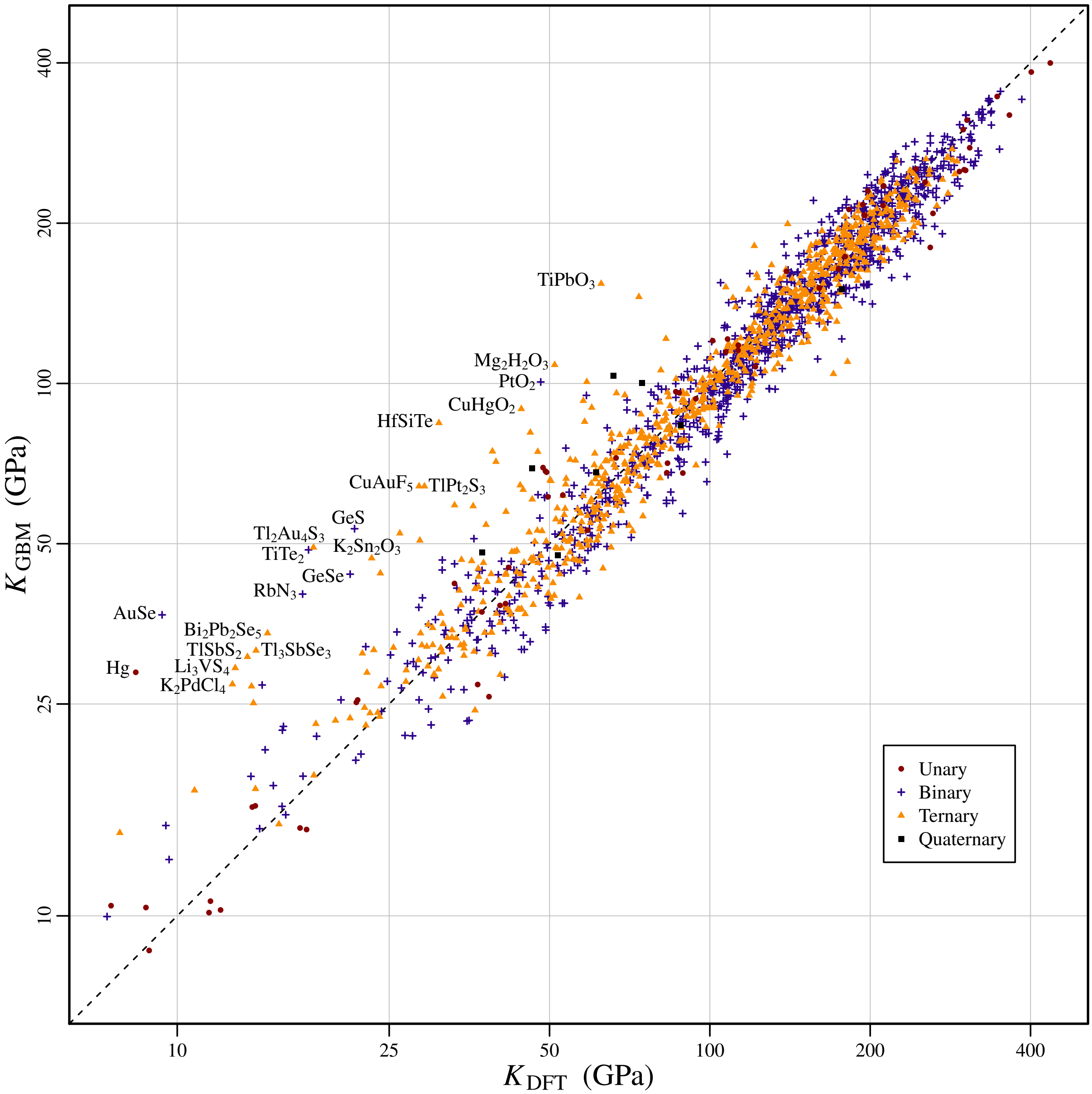

Figure S2: Comparison of K from DFT training set with GBM-Locfit predictions. Training set consists of 65 unary, 1091 binary, 776 ternary, and 8 quaternary compounds. The 20 compounds with largest relative error are labeled. See Table **SII** for Materials Project IDs for these 20 compounds.

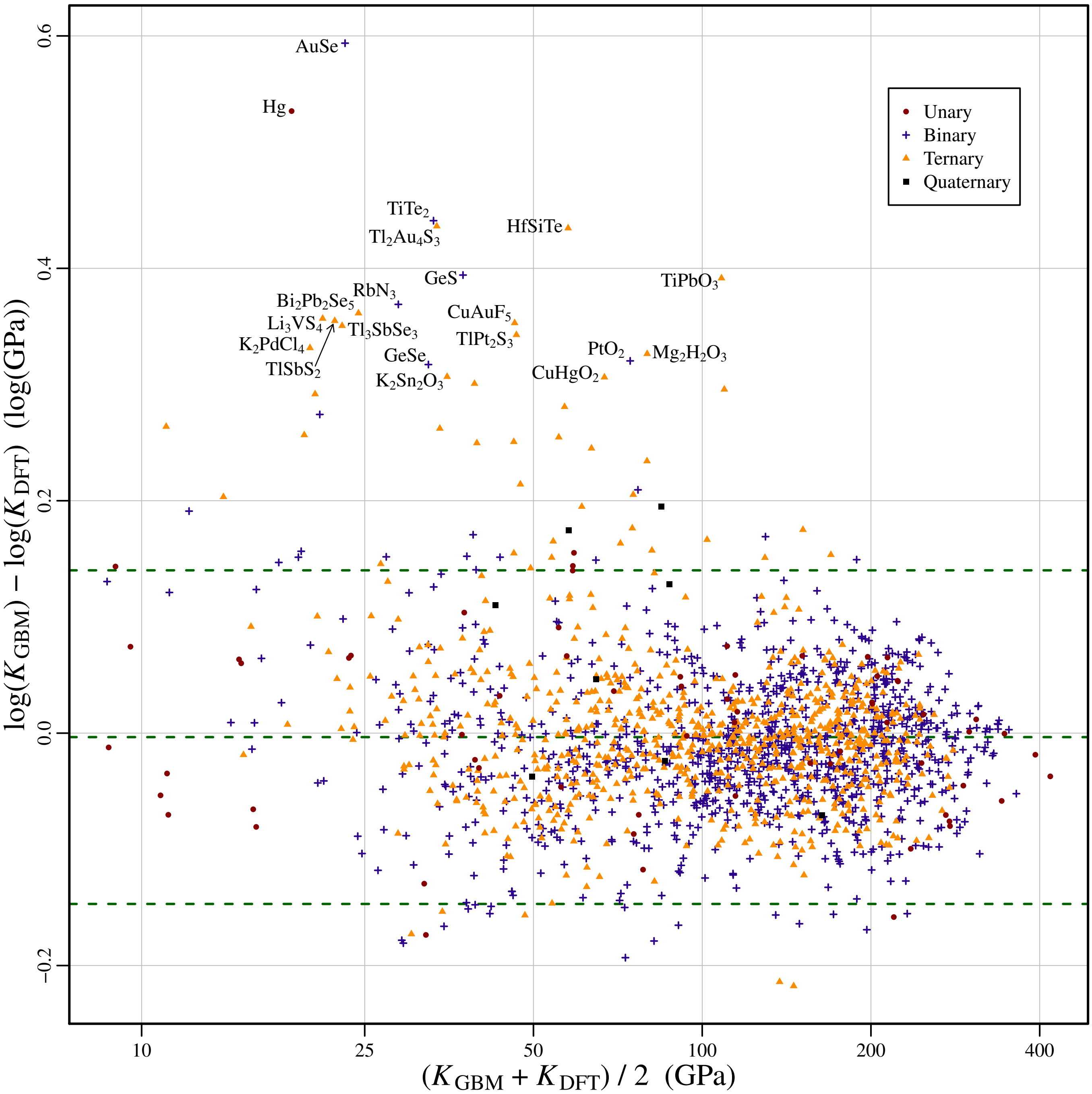

Figure S3: Bland-Altman comparison of K from DFT training set with GBM-Locfit predictions. The three dashed green lines are the mean of the residuals at 0.0034 log(GPa) and the 95% limits of agreement at -0.1401 and 0.1469 log(GPa). The 20 compounds with largest relative error are labeled. See Table **SII** for Materials Project IDs for these 20 compounds.

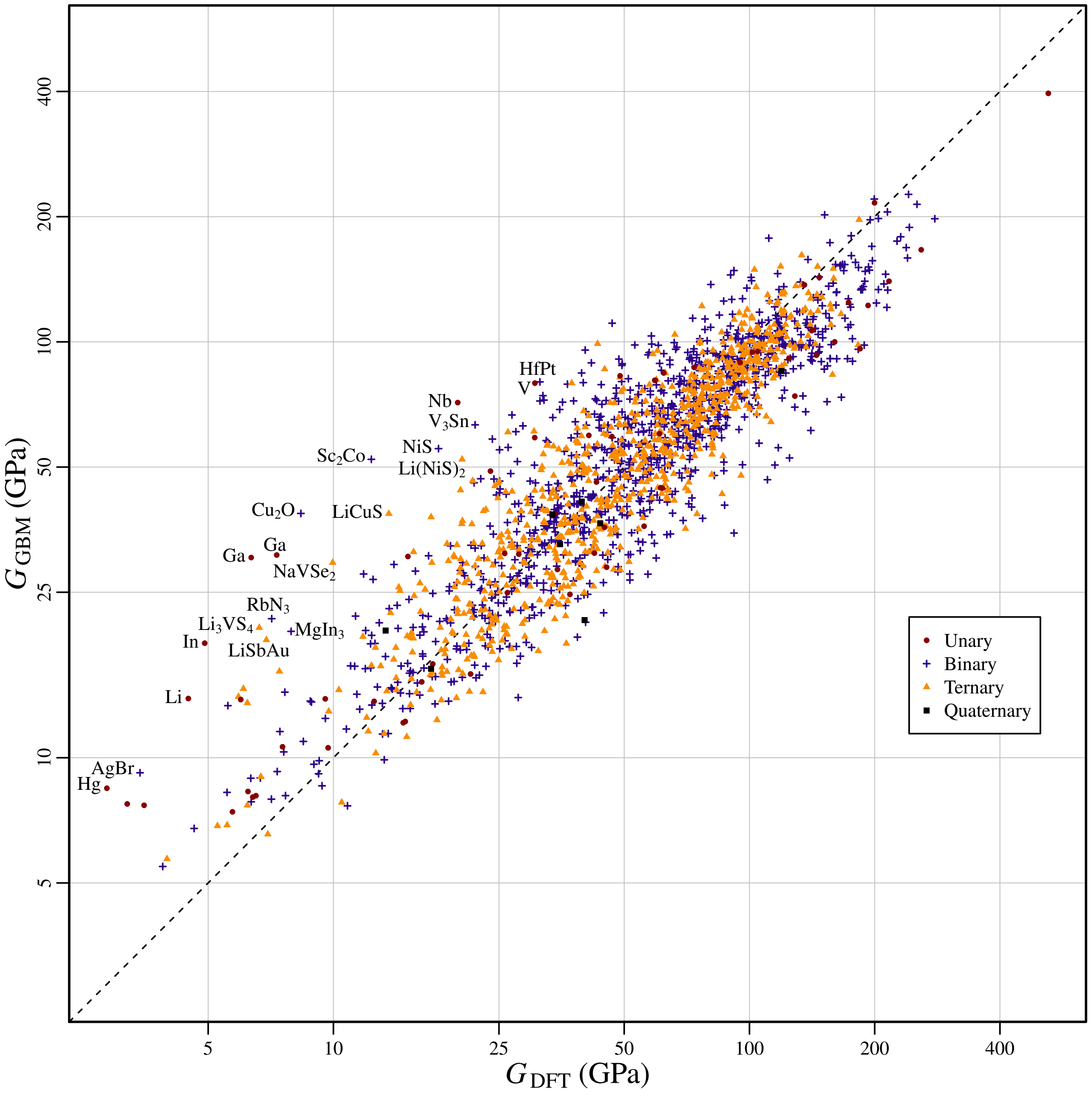

Figure S4: Comparison of G from DFT training set with GBM-Locfit predictions. Training set consists of 65 unary, 1091 binary, 776 ternary, and 8 quaternary compounds. The 20 compounds with largest relative error are labeled. See Table **SII** for Materials Project IDs for these 20 compounds.

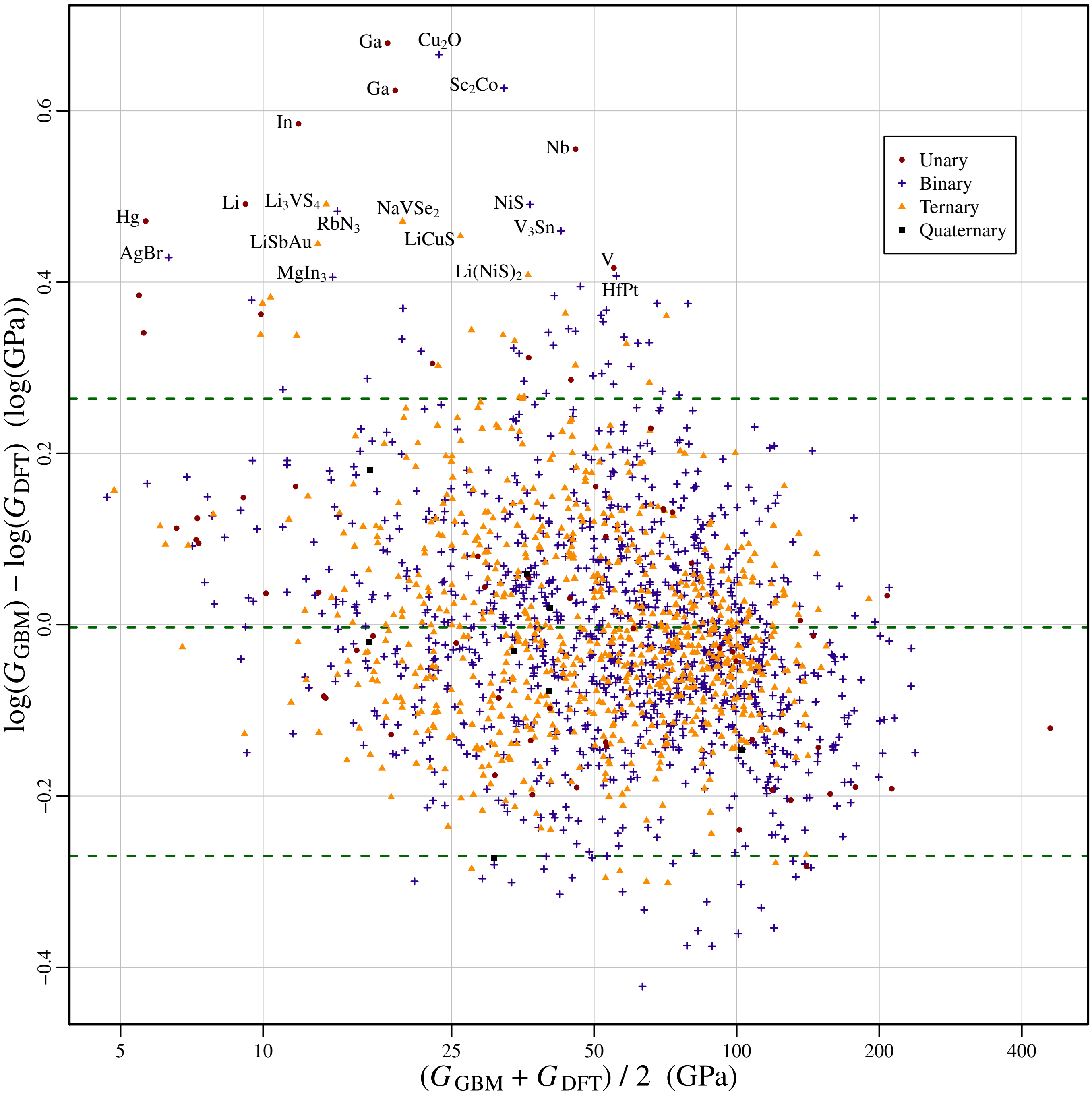

Figure S5: Bland-Altman comparison of G from DFT training set with GBM-Locfit predictions. The three dashed green lines are the mean of the residuals at -0.0031 log(GPa) and the 95% limits of agreement at -0.2699 and 0.2637 log(GPa). The 20 compounds with largest relative error are labeled. See Table **SII** for Materials Project IDs for these 20 compounds.

TABLE SII: **Summary of largest relative errors.** Formula and Materials Project (MP) ID for the 30 compounds with largest relative error for our best four descriptor models for K and G.

Model	Rank	Relative Error (%)	Formula	MP ID
K	1	292.4	AuSe	mp-2793
	2	243.0	Hg	mp-569289
	3	176.1	$TiTe_2$	mp-1907
	4	173.1	$Tl_2Au_4S_3$	mp-29898
	5	172.0	HfSiTe	mp-13963
	6	147.9	GeS	mp-2242
	7	146.4	$TiPbO_3$	mp-20459
	8	133.9	RbN_3	mp-581833
	9	129.8	$Bi_2Pb_2Se_5$	mp-570930
	10	127.4	Li_3VS_4	mp-760375
	11	126.4	$TlSbS_2$	mp-676540
	12	125.4	$CuAuF_5$	mp-28376
	13	124.2	Tl_3SbSe_3	mp-4876
	14	120.2	$TlPt_2S_3$	mp-9272
	15	114.6	K_2PdCl_4	mp-22956
	16	112.0	$Mg_2H_2O_3$	mp-30244
	17	109.1	PtO_2	mp-617
	18	107.6	GeSe	mp-700
	19	102.7	$K_2Sn_2O_3$	mp-8624
	20	102.4	$CuHgO_2$	mp-772856
	21	99.9	K_2AlF_5	mp-9486
	22	97.6	$AlHO_2$	mp-625055
	23	95.8	Bi_2Te_2Se	mp-29666
	24	90.9	VCu_3S_4	mp-3762
	25	88.0	Ga_2Te_5	mp-2371
	26	83.6	Cs_2TeI_6	mp-540957
	27	82.9	$Rb_2Sn_2O_3$	mp-7863
	28	80.5	Tl_2PdCl_4	mp-29889
	29	79.7	$NbCu_3S_4$	mp-5621
	30	78.1	$NbCu_3Se_4$	mp-4043
G	1	377.5	Ga	mp-140
	2	363.0	Cu_2O	mp-361
	3	323.0	Sc_2Co	mp-30562
	4	320.5	Ga	mp-569423
	5	284.4	In	mp-85
	6	259.1	Nb	mp-75
	7	209.8	Li	mp-135
	8	209.7	Li_3VS_4	mp-760375
	9	209.5	NiS	mp-594
	10	203.9	RbN_3	mp-581833
	11	195.9	Hg	mp-569289
	12	195.7	$NaVSe_2$	mp-3567
	13	188.3	V_3Sn	mp-21342
	14	184.2	LiCuS	mp-774712
	15	178.3	LiSbAu	mp-12564
	16	168.3	AgBr	mp-866291
	17	160.9	V	mp-146
	18	155.9	$Li(NiS)_2$	mp-755570
	19	155.4	HfPt	mp-11455
	20	154.4	$MgIn_3$	mp-20566
	21	148.3	Ta_3Sn	mp-30871
	22	142.4	Na	mp-127
	23	142.2	CaC_2	mp-684668
	24	141.1	$TlSbS_2$	mp-676540
	25	139.3	AuSe	mp-2793
	26	137.2	YC_2	mp-313
	27	137.2	KTl_2Bi	mp-866169
	28	137.1	BPt	mp-2192
	29	134.0	Li_3Pd	mp-11489
	30	132.9	Fe_2Ge	mp-20432

Supplementary comments regarding Calfa and Kitchin

Recent work by Calfa and Kitchin (C&K) [10] includes predictions of the Voigt and Reuss polycrystalline limits for K and G, while our work predicts the Hill average of these limits [11]. C&K use a training set of 1,173 crystalline compounds from the Materials Project [12], while we use a larger training set of 1,940 compounds from the same source. More importantly, there are salient methodological differences between the two approaches. First, while our descriptors are capable of "uniquely characterizing" [14] a diverse range of compounds, C&K's descriptors can not distinguish between polymorphs that belong to the same space group. Second, C&K use *categorical* descriptors, while we use *continuous* descriptors. Finally, C&K use *leave-one-out*, or *delete-one*, cross-validation to set kernel bandwidths for smoothing across both descriptors and categories (see equation (4) in [10]), while we use *10-fold* cross-validation to limit the number of GBM iterations. Although both approaches make use of regression and cross-validation in some form, the methodological differences lead to significant generalizability differences between the resulting models.

Although space groups describe the spatial configuration of atoms in each compound, neither space group nor element counts provide sufficient information regarding the strength of interatomic bonds to reliably predict elastic constants. Thus, C&K's descriptors cannot distinguish between polymorphs that belong to the same space group, including some hexagonal graphite and hexagonal diamond allotropes of carbon. As shown in Fig. **S6**, C&K's published model severely under predicts K_{Reuss} for a hexagonal diamond material (mp-611426) from our original dataset [13], as this compound is predicted to have the same modulus as a hexagonal graphite material (mp-48) with the same space group. Additionally, adding this hexagonal diamond material to the training data and generating a new model results in identical predictions for these two carbon allotropes, since they have the same chemical formula and space group, and are thus indistinguishable with C&K's descriptors. Also, the inclusion of this hexagonal diamond material triggers a ripple of reduced prediction accuracy, within the training set itself.

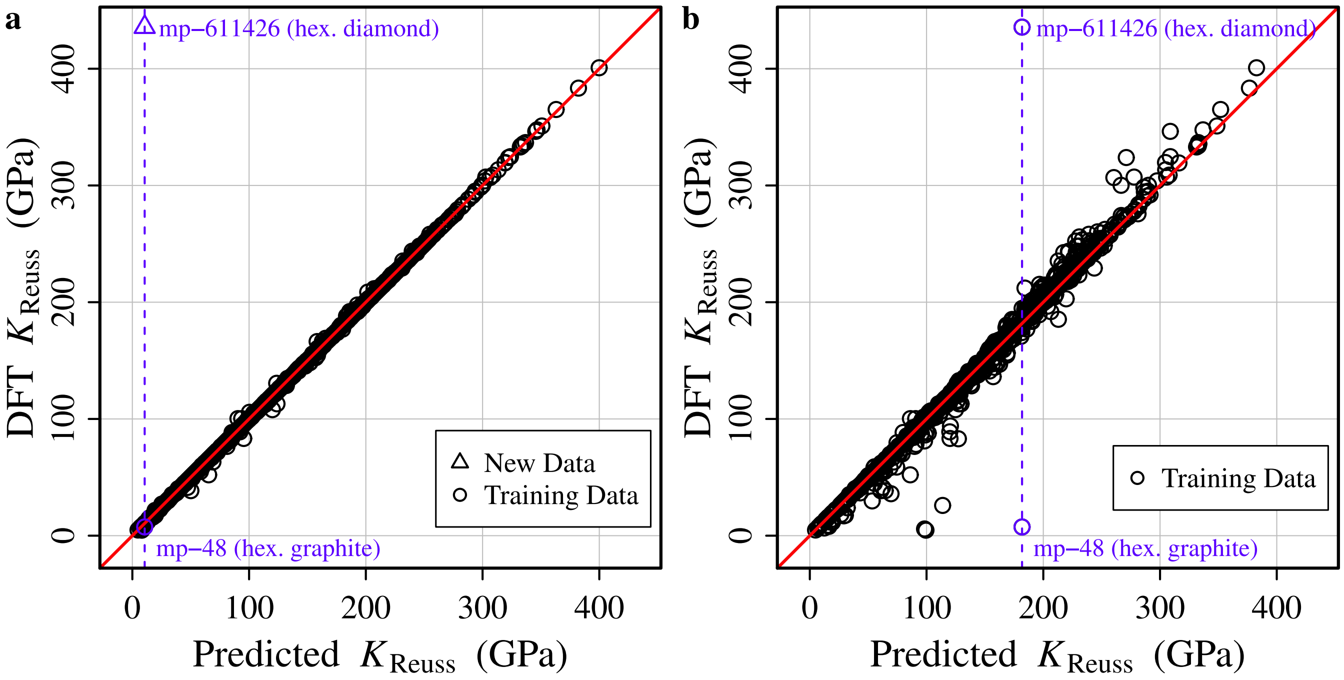

Figure S6: Calfa and Kitchin's published model (a) severely under-predicts the modulus of a hexagonal diamond material (mp-611426), which was not included in their training data, but which has the same space group as a hexagonal graphite material (mp-48). Adding the hexagonal diamond material (mp-611426) to the training data results in a new model (b) that predicts intermediate moduli values for these two carbon allotropes and triggers a ripple of reduced prediction accuracy within the training set itself.

In Fig. **S7** we use C&K's published model to predict K_{Reuss} for 861 compounds in our training set that are not in C&K's training set. The prediction root mean squared error (RMSE) of this sample is 60.9 GPa, which is 48 times larger than C&K's reported prediction RMSE of 1.27 GPa (based on their training set), which suggests their model is significantly over-fit. So although C&K report better prediction accuracy than we do, their reported accuracy fails to generalize to new compounds.

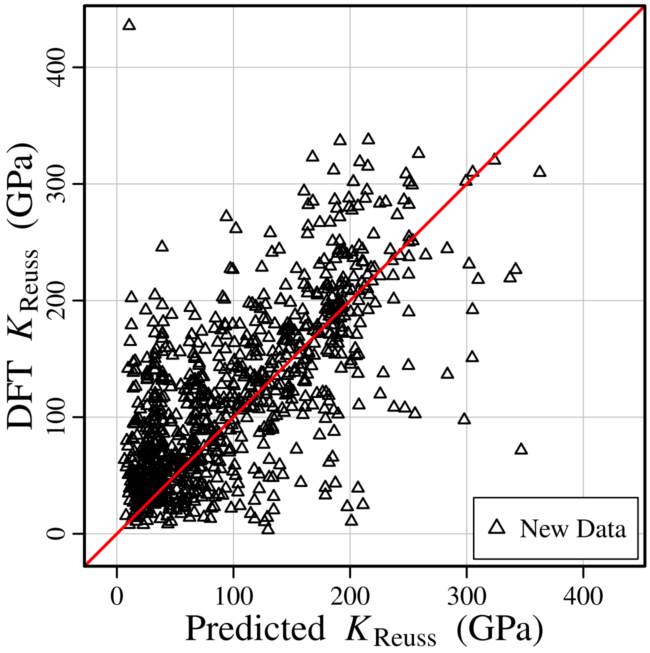

Figure S7: Comparison of DFT data and predictions from Calfa and Kitchin's published model for 861 compounds that are not in their training set. The prediction root mean squared error (RMSE) of this sample is 60.9 GPa, which is 48 times larger than C&K's reported prediction RMSE of 1.27 GPa (based on their training set), which suggests their model is significantly over-fit.

Supplementary screening details

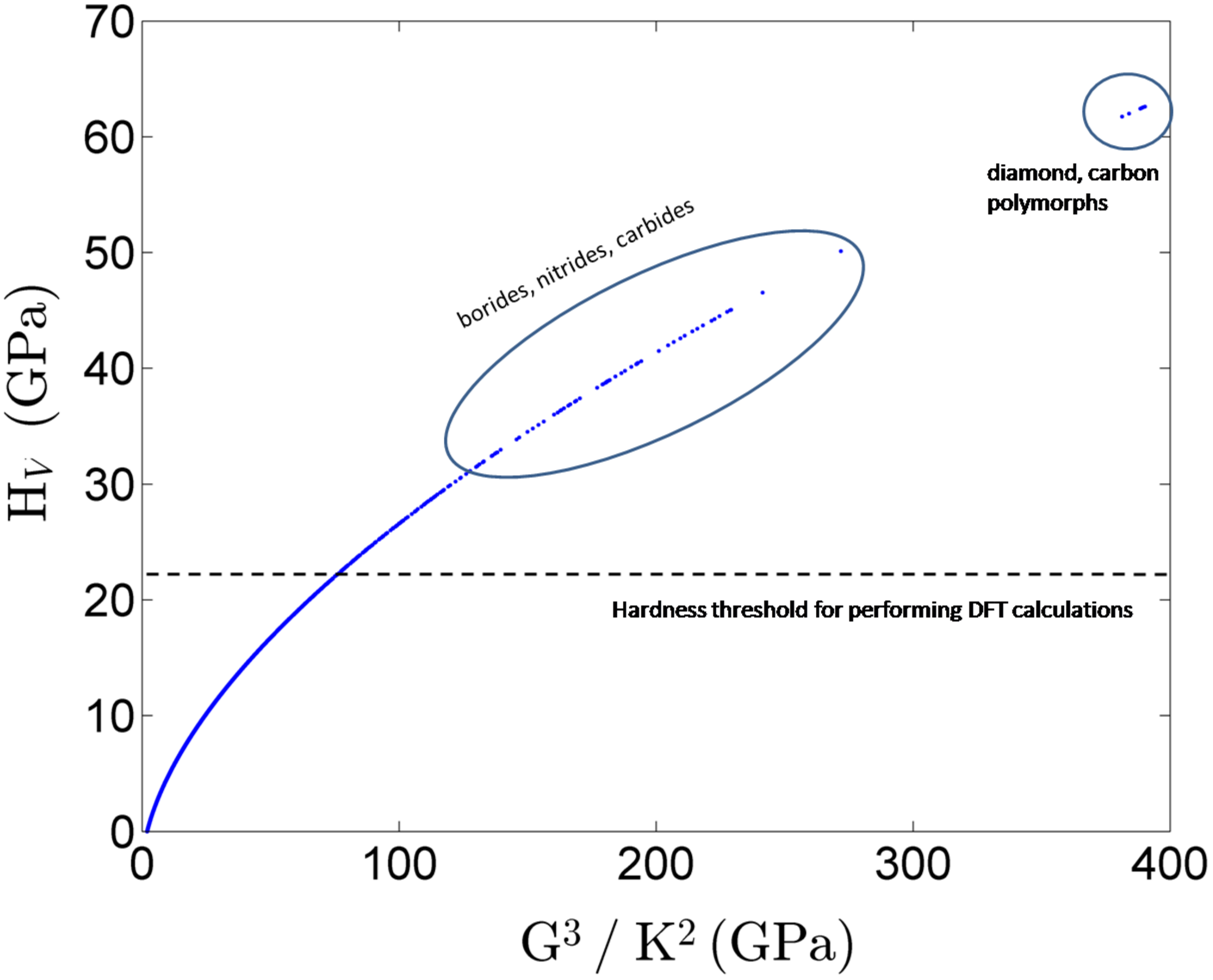

Figure S8: An overview of the predicted hardness from the SL model. The compounds plotted here are those within the set of 30,000 that do not have DFT data available.

TABLE SIII: Overview of the systems selected for a detailed DFT investigation related to high K, G and K/G.

MP-ID	Chemical System	MP-ID	Chemical System	MP-ID	Chemical System
mp-972323	$TaNbTc_2$	mp-867123	$TaTiOs_2$	mp-979291	$TaZnOs_2$
mp-1915	Fe_2B	mp-865482	VTc_2Ge	mp-11507	Ni_4Mo
mp-865048	$TiZnIr_2$	mp-1890	BMo	mp-974616	$RhPt_3$
mp-980109	$SnAu_3$	mp-867779	Ta_2CrFe	mp-977357	Ta_2ReRu
mp-865998	$HfGaIr_2$	mp-865805	$TaZnCo_2$	mp-974358	$RuIr_3$
mp-866134	VFe_3	mp-865144	$CdAu_3$	mp-9973	VB
mp-975834	$MoRu_3$	mp-569270	V_3B_4	mp-11388	GaIr
mp-867792	$ReIr_3$	mp-66	C	mp-867141	$ReOs_3$
mp-864877	Hf_2CoRe	mp-974341	$RuRh_3$	mp-867507	$TaGaFe_2$
mp-867846	$TaTiRe_2$	mp-864640	Ta_2NbIr	mp-972209	Ta_2TcW
mp-864950	$MnAlIr_2$	mp-864798	$HfZnIr_2$	mp-865490	V_2CrFe
mp-866375	$TiFe_2Ge$	mp-20787	FeB	mp-979751	$TaTiFe_2$
mp-973834	$PdAu_3$	mp-977541	Hf_2TcOs	mp-865497	V_2ReOs
mp-1077	B_2Ru	mp-15671	Re_3B	mp-867816	$TaRu_3$
mp-569516	$HfNi_5$	mp-7573	$Co(BW)_2$	mp-975065	Re
mp-1867	Ta_2Ni	mp-865804	$TaZnRh_2$	mp-2536	Ni_2B
mp-30708	$HfNi_2$	mp-865094	$HfNbTc_2$	mp-891	$TaNi_3$
mp-862621	$GaPt_3$	mp-973839	$PdAu_3$	mp-979910	$VCuRh_2$
mp-865656	$TiMn_2W$	mp-1010	MnB_4	mp-865045	$MnRu_3$
mp-81	Au	mp-865494	V_2CrRu	mp-493	Co_2B
mp-867345	$TcIr_3$	mp-10257	Ni	mp-867264	Re_3Os
mp-861626	Tc_3Rh	mp-974395	$RhPt_3$	mp-676416	V_2CN
mp-1097	TaB	mp-865485	V_2CrOs	mp-864764	Ta_2FeOs
mp-864985	$MnZnIr_2$	mp-30555	$TaGaCo_2$	mp-15703	$BeCN_2$
mp-38818	$HfNbB_4$	mp-974455	Re_3Ru	mp-977407	Hf_2ReRh
mp-979288	Ta_2NbRh	mp-862705	$AlZnIr_2$	mp-865484	V_2CrRe
mp-864770	Ta_2MoOs	mp-1113	BW_2	mp-865038	$HfTaTc_2$
mp-2213	FeNi	mp-865495	V_2CrTc	mp-11513	$NbNi_3$
mp-865179	Hf_2FeOs	mp-21244	Cr_3RhN	mp-974625	Re_3Ru
mp-866142	$TiGaIr_2$	mp-9999	$Ni(BMo)_2$	mp-569683	$InAu_3$
mp-862620	Ru_3Ir	mp-10142	Ta_3B_4	mp-864670	Ta_2VOs
mp-977390	Ta_3Ru	mp-30811	Ni_4W	mp-865653	$TiNbRe_2$
mp-866127	$LuTaOs_2$	mp-977426	$NbCo_3$	mp-977425	Ta_2CrRu
mp-982070	VIr	mp-974421	RuIr	mp-867865	$ReRh_3$
mp-867327	Hf_2TcRu	mp-864650	Ta_2OsW	mp-867788	$TaGaOs_2$
mp-1761	$LuIr_2$	mp-864651	Ta_2NbRu	mp-974437	Re_2C
mp-1132	CdO	mp-889	Cr_3B_4	mp-7832	BW
mp-865234	$TaZnRu_2$	mp-865088	$MnGaIr_2$	mp-863709	Ir_3Rh
mp-862770	Tc_3Ir	mp-867916	$ScGaIr_2$	mp-978532	$SiCuRh_2$
mp-865385	V_3Re	mp-861630	Tc_3Ru	mp-867356	$TcRu_3$
mp-10596	$FeIr_3$	mp-972326	$TcPt_3$	mp-570863	$CrCoPt_2$
mp-1438	$ZrIr_3$	mp-2439	$ZrNi_5$	mp-864835	Hf_2CoIr
mp-10055	Co_2B_4Mo	mp-16315	$GePt_3$	mp-2082	PIr_2
mp-867958	$TaInRu_2$	mp-865465	VCo_2Ge	mp-1773	ReB_2
mp-974492	$ReTc_3$	mp-27710	CrB_4	mp-974430	Re_3Ir
mp-974376	Rh_3Pt	mp-979289	TaW_3	mp-864761	Ta_2MnOs
mp-864983	$MnVRu_2$	mp-977356	$ZnGaIr_2$	mp-15722	Fe_2B_4Mo
mp-974370	Rh_3Pt	mp-977353	Ta_2ReMo	mp-974435	Re_3N
mp-974411	Re_3Tc	mp-867303	$AgAu_3$	mp-862630	$ReRu_3$
mp-867212	$TcOs_3$	mp-972220	$TiPt_3$	mp-12110	TaRu
mp-977454	$HgPd_2Au$	mp-2850	B_2Os	mp-1451	$NbNi_3$
mp-865506	$VSiOs_2$	mp-862779	Tc_3Os	mp-29752	$NiGePt_2$
mp-971754	V_2ReW	mp-865025	$HfTaRe_2$	mp-865022	$MnIr_3$
mp-865916	$TiVRe_2$	mp-979267	Ta_2FeMo	mp-867774	Ta_2CrOs
mp-1279	TaN	mp-15226	$CrWN_2$	mp-260	CrB
mp-862609	$ScReTc_2$	mp-985287	$AgAu_3$	mp-864735	Mn_3Ir
mp-864684	Hf_2MoIr	mp-21175	$TaGaNi_2$	mp-974326	Ru_3Os
mp-864890	Hf_2FeIr	mp-790	$LuPt_3$		

TABLE SIV: Top 60 systems with highest bulk modulus according to DFT, sorted in descending order by bulk modulus. Compounds and elements that were used in the training of the statistical learning model are excluded from the table in order to show only systems that were truly discovered by statistical learning. This is the reason that for example osmium (Os) is not included, although it has a very high bulk modulus of 401 GPa.

MP-ID	Chemical System	K_{DFT} (GPa)	K_{ML} (GPa)	G_{DFT} (GPa)	G_{ML} (GPa)	K/G (DFT)	K/G (ML)
mp-66	C (diamond)	435.7	400.6	520.3	397.1	0.84	1.01
mp-974437	Re_2C	399.3	362.3	262.0	167.4	1.52	2.16
mp-867141	$ReOs_3$	394.8	369.9	255.8	159.1	1.54	2.32
mp-974435	Re_3N	388.2	342.6	206.0	129.7	1.88	2.64
mp-867212	$TcOs_3$	378.6	350.2	245.1	152.2	1.54	2.30
mp-867264	Re_3Os	376.5	335.5	208.4	137.1	1.81	2.45
mp-975065	Re	367.1	318.2	169.2	123.9	2.17	2.57
mp-974430	Re_3Ir	364.5	326.8	212.2	130.7	1.72	2.50
mp-15671	Re_3B	360.9	348.6	259.7	151.7	1.39	2.30
mp-867792	$ReIr_3$	357.4	344.9	150.4	140.6	2.38	2.45
mp-974455	Re_3Ru	355.3	312.9	187.4	127.1	1.90	2.46
mp-974625	Re_3Ru	351.7	314.4	190.4	127.4	1.85	2.47
mp-974411	Re_3Tc	348.4	304.3	166.3	119.5	2.10	2.55
mp-570863	$CrCoPt_2$	346.5	237.7	113.4	80.4	3.06	2.96
mp-867345	$TcIr_3$	342.3	327.4	147.8	135.6	2.32	2.41
mp-7832	BW	342.2	333.8	194.0	184.5	1.76	1.81
mp-7573	$Co(BW)_2$	341.2	329.5	207.0	178.7	1.65	1.84
mp-1773	ReB_2	336.6	325.3	269.5	201.9	1.25	1.61
mp-974358	$RuIr_3$	336.5	332.5	218.8	137.3	1.54	2.42
mp-974326	Ru_3Os	332.8	308.7	209.3	135.1	1.59	2.29
mp-1113	BW_2	330.2	337.4	145.0	153.7	2.28	2.19
mp-974421	RuIr	329.7	317.0	161.9	133.0	2.04	2.38
mp-862630	$ReRu_3$	327.8	298.1	201.2	128.8	1.63	2.31
mp-862779	Tc_3Os	325.2	286.1	175.7	122.2	1.85	2.34
mp-862620	Ru_3Ir	322.1	300.9	184.1	129.4	1.75	2.33
mp-863709	Ir_3Rh	321.8	320.6	201.5	130.2	1.60	2.46
mp-974492	$ReTc_3$	316.3	273.3	152.9	111.3	2.07	2.46
mp-15703	$BeCN_2$	315.4	249.4	292.6	191.9	1.08	1.30
mp-867356	$TcRu_3$	313.2	273.7	197.1	121.5	1.59	2.25
mp-1890	BMo	312.2	272.1	196.7	155.3	1.59	1.75
mp-862770	Tc_3Ir	311.5	284.5	173.0	120.5	1.80	2.36
mp-2850	B_2Os	310.5	323.6	161.5	196.7	1.92	1.65
mp-1279	TaN	309.8	284.2	186.1	156.7	1.66	1.81
mp-676416	V_2CN	305.4	282.0	190.5	174.5	1.60	1.62
mp-260	CrB	303.7	263.3	217.2	167.2	1.40	1.57
mp-861630	Tc_3Ru	301.2	259.7	165.4	112.9	1.82	2.30
mp-975834	$MoRu_3$	299.2	259.8	162.1	115.3	1.85	2.25
mp-889	Cr_3B_4	298.4	267.0	209.0	179.4	1.43	1.49
mp-15722	Fe_2B_4Mo	297.1	300.9	176.2	189.8	1.69	1.58
mp-9999	$Ni(BMo)_2$	296.2	269.4	177.5	148.1	1.67	1.82
mp-10055	Co_2B_4Mo	295.0	306.1	176.7	193.6	1.67	1.58
mp-865497	V_2ReOs	294.5	274.7	140.2	116.0	2.10	2.37
mp-10142	Ta_3B_4	294.4	302.3	205.8	199.7	1.43	1.51
mp-864735	Mn_3Ir	291.1	238.8	154.6	81.9	1.88	2.92
mp-867865	$ReRh_3$	289.2	269.1	122.6	107.7	2.36	2.50
mp-1097	TaB	286.2	288.3	211.8	184.8	1.35	1.56
mp-861626	Tc_3Rh	284.8	255.0	156.2	110.0	1.82	2.32
mp-865022	$MnIr_3$	284.1	305.7	136.3	117.5	2.08	2.60
mp-20787	FeB	282.9	303.0	139.1	190.9	2.03	1.59
mp-865506	$VSiOs_2$	279.5	274.8	116.1	118.9	2.41	2.31
mp-27710	CrB_4	279.5	255.3	258.3	207.2	1.08	1.23
mp-569270	V_3B_4	279.4	274.9	232.9	203.1	1.20	1.35
mp-867816	$TaRu_3$	279.2	270.4	130.0	122.1	2.15	2.21
mp-1077	B_2Ru	278.6	284.6	170.0	180.9	1.64	1.57
mp-979289	TaW_3	278.0	280.3	123.8	133.6	2.25	2.10
mp-982070	VIr	277.7	271.3	122.6	115.0	2.26	2.36
mp-1010	MnB_4	275.6	253.1	240.3	206.7	1.15	1.22
mp-2082	PIr_2	275.0	248.6	92.2	94.0	2.98	2.64
mp-867788	$TaGaOs_2$	273.7	271.2	126.8	112.3	2.16	2.42
mp-9973	VB	273.3	267.6	232.3	187.6	1.18	1.43

TABLE SV: Top 60 systems with highest shear modulus according to DFT, sorted in descending order by shear modulus. Compounds and elements that were used in the training of the statistical learning model are excluded from the table in order to show only systems that were truly discovered by statistical learning. This is the reason that for example osmium (Os) is not included, although it has a very high shear modulus of 259 GPa.

MP-ID	Chemical System	G_{DFT} (GPa)	G_{ML} (GPa)	K_{DFT} (GPa)	K_{ML} (GPa)	K/G (DFT)	K/G (ML)
mp-66	C (diamond)	520.3	397.1	435.7	400.6	0.84	1.01
mp-865998	$HfGaIr_2$	355.7	92.9	210.9	227.9	0.59	2.45
mp-15703	$BeCN_2$	292.6	191.9	315.4	249.4	1.08	1.30
mp-1773	ReB_2	269.5	201.9	336.6	325.3	1.25	1.61
mp-974437	Re_2C	262.0	167.4	399.3	362.3	1.52	2.16
mp-15671	Re_3B	259.7	151.7	360.9	348.6	1.39	2.30
mp-27710	CrB_4	258.3	207.2	279.5	255.3	1.08	1.23
mp-867141	$ReOs_3$	255.8	159.1	394.8	369.9	1.54	2.32
mp-867212	$TcOs_3$	245.1	152.2	378.6	350.2	1.54	2.30
mp-1010	MnB_4	240.3	206.7	275.6	253.1	1.15	1.22
mp-569270	V_3B_4	232.9	203.1	279.4	274.9	1.20	1.35
mp-9973	VB	232.3	187.6	273.3	267.6	1.18	1.43
mp-974358	$RuIr_3$	218.8	137.3	336.5	332.5	1.54	2.42
mp-260	CrB	217.2	167.2	303.7	263.3	1.40	1.57
mp-38818	$HfNbB_4$	214.9	194.0	269.0	272.2	1.25	1.40
mp-974430	Re_3Ir	212.2	130.7	364.5	326.8	1.72	2.50
mp-1097	TaB	211.8	184.8	286.2	288.3	1.35	1.56
mp-974326	Ru_3Os	209.3	135.1	332.8	308.7	1.59	2.29
mp-889	Cr_3B_4	209.0	179.4	298.4	267.0	1.43	1.49
mp-867264	Re_3Os	208.4	137.1	376.5	335.5	1.81	2.45
mp-7573	$Co(BW)_2$	207.0	178.7	341.2	329.5	1.65	1.84
mp-974435	Re_3N	206.0	129.7	388.2	342.6	1.88	2.64
mp-10142	Ta_3B_4	205.8	199.7	294.4	302.3	1.43	1.51
mp-10596	$FeIr_3$	202.4	127.3	-393.3	330.6	-1.94	2.60
mp-863709	Ir_3Rh	201.5	130.2	321.8	320.6	1.60	2.46
mp-862630	$ReRu_3$	201.2	128.8	327.8	298.1	1.63	2.31
mp-867356	$TcRu_3$	197.1	121.5	313.2	273.7	1.59	2.25
mp-1890	BMo	196.7	155.3	312.2	272.1	1.59	1.75
mp-7832	BW	194.0	184.5	342.2	333.8	1.76	1.81
mp-676416	V_2CN	190.5	174.5	305.4	282.0	1.60	1.62
mp-974625	Re_3Ru	190.4	127.4	351.7	314.4	1.85	2.47
mp-974455	Re_3Ru	187.4	127.1	355.3	312.9	1.90	2.46
mp-1279	TaN	186.1	156.7	309.8	284.2	1.66	1.81
mp-862620	Ru_3Ir	184.1	129.4	322.1	300.9	1.75	2.33
mp-9999	$Ni(BMo)_2$	177.5	148.1	296.2	269.4	1.67	1.82
mp-10055	Co_2B_4Mo	176.7	193.6	295.0	306.1	1.67	1.58
mp-15722	Fe_2B_4Mo	176.2	189.8	297.1	300.9	1.69	1.58
mp-862779	Tc_3Os	175.7	122.2	325.2	286.1	1.85	2.34
mp-862770	Tc_3Ir	173.0	120.5	311.5	284.5	1.80	2.36
mp-865045	$MnRu_3$	170.3	103.1	229.2	250.6	1.35	2.43
mp-1077	B_2Ru	170.0	180.9	278.6	284.6	1.64	1.57
mp-975065	Re	169.2	123.9	367.1	318.2	2.17	2.57
mp-974411	Re_3Tc	166.3	119.5	348.4	304.3	2.10	2.55
mp-861630	Tc_3Ru	165.4	112.9	301.2	259.7	1.82	2.30
mp-975834	$MoRu_3$	162.1	115.3	299.2	259.8	1.85	2.25
mp-974421	RuIr	161.9	133.0	329.7	317.0	2.04	2.38
mp-2850	B_2Os	161.5	196.7	310.5	323.6	1.92	1.65
mp-861626	Tc_3Rh	156.2	110.0	284.8	255.0	1.82	2.32
mp-864735	Mn_3Ir	154.6	81.9	291.1	238.8	1.88	2.92
mp-1438	$ZrIr_3$	153.1	122.8	267.5	271.4	1.75	2.21
mp-974492	$ReTc_3$	152.9	111.3	316.3	273.3	2.07	2.46
mp-867792	$ReIr_3$	150.4	140.6	357.4	344.9	2.38	2.45
mp-867345	$TcIr_3$	147.8	135.6	342.3	327.4	2.32	2.41
mp-21244	Cr_3RhN	146.5	83.9	270.6	207.6	1.85	2.47
mp-15226	$CrWN_2$	146.5	122.6	259.4	283.5	1.77	2.31
mp-1915	Fe_2B	145.1	154.5	223.4	276.6	1.54	1.79
mp-1113	BW_2	145.0	153.7	330.2	337.4	2.28	2.19
mp-865497	V_2ReOs	140.2	116.0	294.5	274.7	2.10	2.37
mp-20787	FeB	139.1	190.9	282.9	303.0	2.03	1.59
mp-865022	$MnIr_3$	136.3	117.5	284.1	305.7	2.08	2.60

TABLE SVI: Top 60 systems with highest Pugh's ratio (K/G) according to DFT, sorted in descending order by K/G.

MP-ID	Chemical System	K/G (DFT)	K/G (ML)	K_{DFT} (GPa)	K_{ML} (GPa)	G_{DFT} (GPa)	G_{ML} (GPa)
mp-980109	$SnAu_3$	9.9	4.0	97.7	103.4	9.91	25.91
mp-973834	$PdAu_3$	7.5	4.3	142.1	136.0	19.05	31.58
mp-867303	$AgAu_3$	7.0	4.4	124.0	121.1	17.78	27.31
mp-973839	$PdAu_3$	6.4	4.3	142.2	135.4	22.22	31.49
mp-569683	$InAu_3$	6.3	4.2	102.8	110.2	16.32	26.28
mp-985287	$AgAu_3$	5.8	4.4	122.2	122.3	21.01	27.52
mp-865144	$CdAu_3$	5.3	4.5	111.7	109.3	21.04	24.02
mp-865385	V_3Re	4.6	2.4	237.6	220.1	52.10	92.81
mp-21175	$TaGaNi_2$	4.2	2.6	145.3	213.7	34.98	81.06
mp-979910	$VCuRh_2$	3.8	2.6	223.2	210.9	58.36	82.47
mp-11388	GaIr	3.7	2.8	214.0	225.3	58.40	80.25
mp-974395	$RhPt_3$	3.6	2.9	251.2	251.0	70.20	85.24
mp-862621	$GaPt_3$	3.4	3.0	211.8	227.4	63.03	74.88
mp-974616	$RhPt_3$	3.3	2.9	242.9	250.8	72.83	85.17
mp-864764	Ta_2FeOs	3.1	2.4	249.8	263.8	81.47	111.76
mp-29752	$NiGePt_2$	3.1	2.9	200.5	223.8	65.55	78.26
mp-570863	$CrCoPt_2$	3.1	3.0	346.5	237.7	113.37	80.38
mp-2082	PIr_2	3.0	2.6	275.0	248.6	92.20	94.01
mp-978532	$SiCuRh_2$	3.0	2.5	218.8	207.9	74.01	81.87
mp-979291	$TaZnOs_2$	2.9	2.6	258.2	256.6	89.57	99.46
mp-864761	Ta_2MnOs	2.9	2.5	234.7	259.2	81.71	105.40
mp-979751	$TaTiFe_2$	2.9	2.4	199.0	216.2	69.72	90.44
mp-865805	$TaZnCo_2$	2.8	2.8	207.7	211.5	73.85	76.85
mp-864890	Hf_2FeIr	2.8	2.4	188.7	205.9	67.42	85.29
mp-864985	$MnZnIr_2$	2.8	3.0	225.9	229.5	81.73	76.71
mp-1132	CdO	2.7	4.3	127.6	107.2	46.43	25.20
mp-867123	$TaTiOs_2$	2.7	2.2	268.6	275.3	100.10	123.67
mp-864670	Ta_2VOs	2.7	2.3	248.0	255.8	93.16	110.34
mp-865482	VTc_2Ge	2.6	2.4	236.5	212.2	89.26	89.02
mp-865495	V_2CrTc	2.6	2.4	241.3	203.9	91.79	84.46
mp-865234	$TaZnRu_2$	2.6	2.5	225.9	221.7	86.13	88.22
mp-493	Co_2B	2.6	1.8	244.3	292.7	95.09	161.86
mp-864983	$MnVRu_2$	2.5	2.4	238.4	216.2	94.19	89.74
mp-972220	$TiPt_3$	2.5	2.6	234.5	246.6	92.83	96.25
mp-2536	Ni_2B	2.5	1.9	243.7	286.0	96.48	154.52
mp-12110	TaRu	2.5	2.2	244.4	253.8	97.25	113.30
mp-10257	Ni	2.5	2.6	196.4	228.5	78.16	88.46
mp-1867	Ta_2Ni	2.5	2.4	207.1	238.8	82.56	97.58
mp-977390	Ta_3Ru	2.5	2.3	230.4	239.2	92.15	102.64
mp-11513	$NbNi_3$	2.5	2.5	218.3	225.5	87.94	91.52
mp-974341	$RuRh_3$	2.5	2.5	267.1	247.0	108.26	98.50
mp-1451	$NbNi_3$	2.5	2.5	202.5	226.1	82.62	91.81
mp-865094	$HfNbTc_2$	2.4	2.3	217.9	204.1	89.22	87.16
mp-866142	$TiGaIr_2$	2.4	2.5	222.1	233.3	90.94	94.64
mp-864684	Hf_2MoIr	2.4	2.3	191.1	202.1	78.34	86.66
mp-865494	V_2CrRu	2.4	2.4	245.6	207.6	100.89	86.76
mp-865465	VCo_2Ge	2.4	2.4	177.5	202.1	72.98	84.71
mp-972209	Ta_2TcW	2.4	2.2	252.0	253.7	103.66	114.70
mp-864651	Ta_2NbRu	2.4	2.3	225.9	227.4	93.32	98.69
mp-865916	$TiVRe_2$	2.4	2.4	254.6	247.9	105.50	103.32
mp-790	$LuPt_3$	2.4	2.5	185.9	214.3	77.06	86.09
mp-11507	Ni_4Mo	2.4	2.6	214.3	232.3	88.84	89.52
mp-867327	Hf_2TcRu	2.4	2.3	192.7	205.6	79.93	88.41
mp-865506	$VSiOs_2$	2.4	2.3	279.5	274.8	116.14	118.88
mp-891	$TaNi_3$	2.4	2.5	209.4	258.8	87.51	101.78
mp-977425	Ta_2CrRu	2.4	2.4	237.0	239.2	99.37	99.74
mp-867792	$ReIr_3$	2.4	2.5	357.4	344.9	150.39	140.65
mp-867916	$ScGaIr_2$	2.4	2.4	193.5	209.7	81.47	86.21
mp-864640	Ta_2NbIr	2.4	2.3	236.7	239.0	99.69	102.80
mp-865653	$TiNbRe_2$	2.4	2.4	254.9	234.4	107.37	98.43

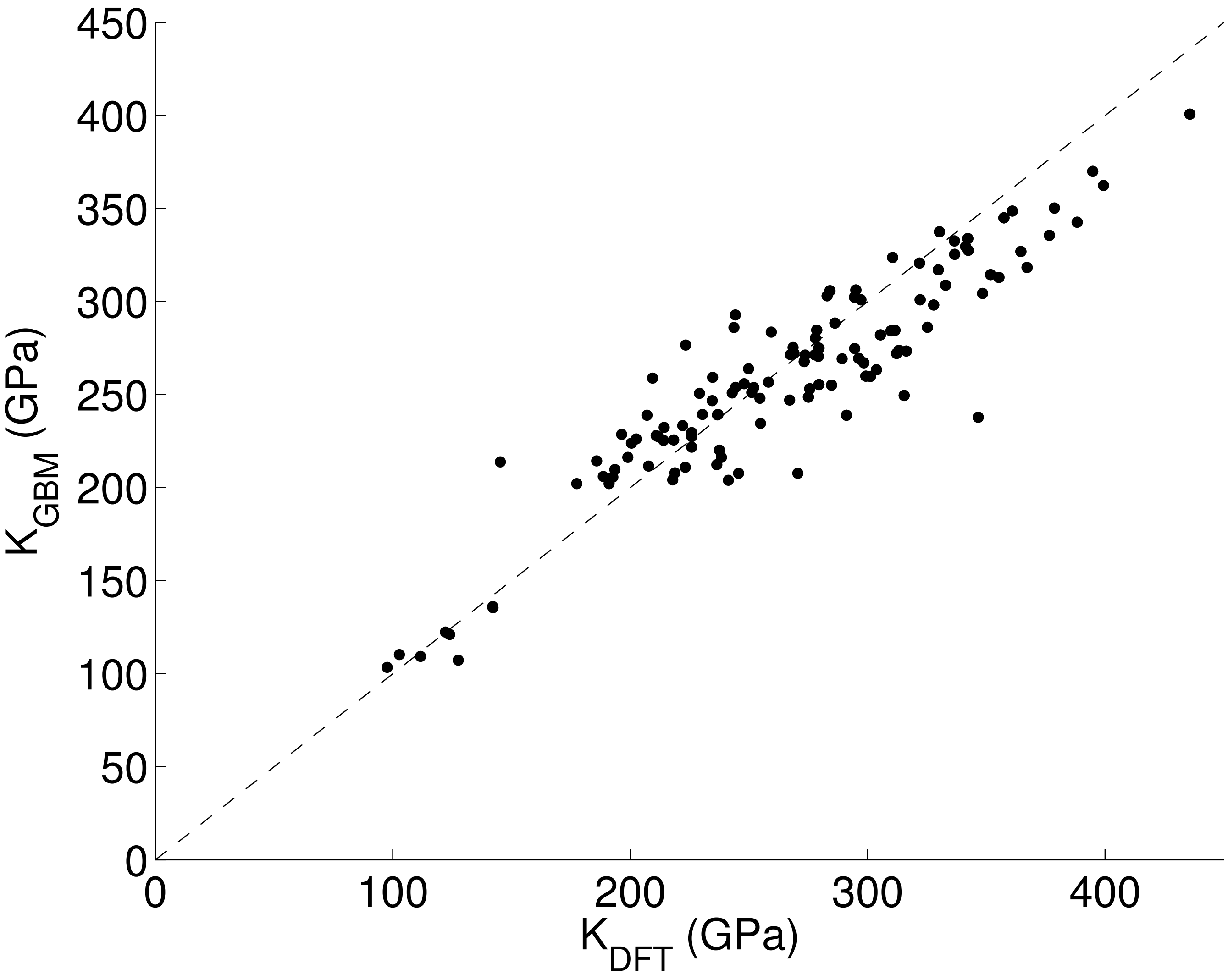

Figure S9: Comparison of K_{DFT} and K_{GBM} for the compounds listed in Table **SIII**.

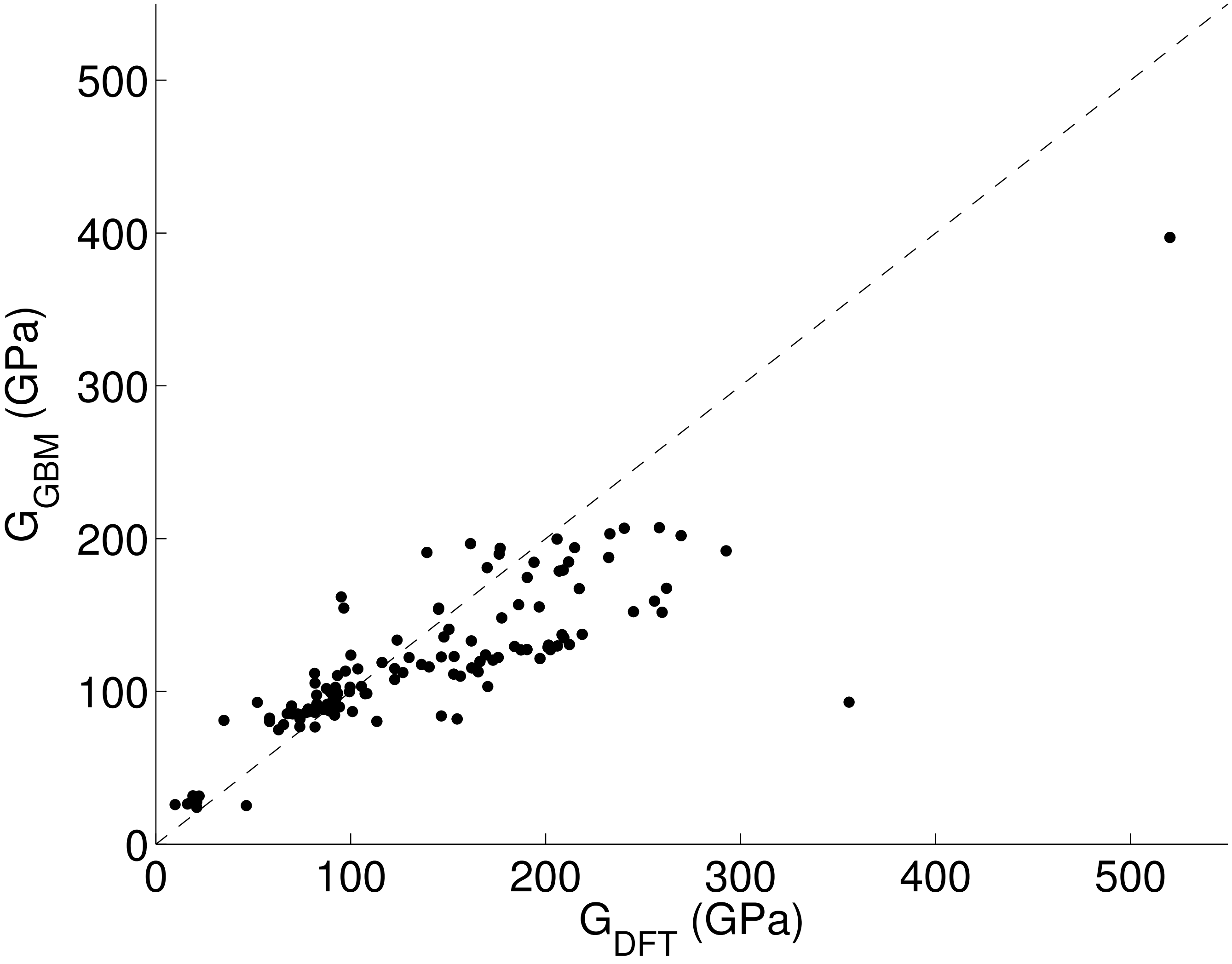

Figure S10: Comparison of G_{DFT} and G_{GBM} for the compounds listed in Table **SIII**.

Supplementary References

1. Friedman, J. H. Greedy function approximation: A gradient boosting machine. *Annals of Statistics* **29**, 1189–1232 (2001).
2. Barron, A., Birgé, L. & Massart, P. Risk bounds for model selection via penalization. *Probability Theory and Related Fields* **113**, 301–413 (1999).
3. Efron, B. The estimation of prediction error. *Journal of the American Statistical Association* **99** (2004).
4. Copas, J. B. Regression, prediction and shrinkage. *Journal of the Royal Statistical Society. Series B (Methodological)* 311–354 (1983).
5. Hastie, T., Tibshirani, R. & Friedman, J. *The elements of statistical learning: data mining, inference, and prediction* 364–365 (Springer, 2011), second edn.
6. Breiman, L., Friedman, J. H., Olshen, R. A. & Stone, C. J. *Classification and regression trees* 55–58 (CRC press, 1993).
7. Breiman, L. Random forests. *Machine Learning* **45**, 5–32 (2001).
8. Loader, C. *Local regression and likelihood*, vol. 47 (Springer New York, 1999).
9. Ku, H.-T., Ku, M.-C. & Zhang, X.-M. Generalized power means and interpolating inequalities. *Proceedings of the American Mathematical Society* **127**, 145–154 (1999).
10. Calfa, B. A. & Kitchin, J. R. Property prediction of crystalline solids from composition and crystal structure. *AIChE Journal* (2016).
11. Hill, R. The elastic behaviour of a crystalline aggregate. *Proceedings of the Physical Society. Section A* **65**, 349 (1952).
12. Jain, A. *et al.* Commentary: The materials project: A materials genome approach to accelerating materials innovation. *Apl Materials* **1**, 011002 (2013).
13. de Jong, M. *et al.* Charting the complete elastic properties of inorganic crystalline compounds. *Scientific Data* **2** (2015).
14. Ghiringhelli, L. M., Vybiral, J., Levchenko, S. V., Draxl, C. & Scheffler, M. Big data of materials science: Critical role of the descriptor. *Physical Review Letters* **114**, 105503 (2015).
